# Mixed-linker strategy for suppressing structural flexibility of metal-organic framework membranes for gas separation

**DOI:** 10.1038/s42004-023-00917-2

**Published:** 2023-06-10

**Authors:** Chung-Kai Chang, Ting-Rong Ko, Tsai-Yu Lin, Yen-Chun Lin, Hyun Jung Yu, Jong Suk Lee, Yi-Pei Li, Heng-Liang Wu, Dun-Yen Kang

**Affiliations:** 1 Department of Chemical Engineering, National Taiwan University, No. 1, Sec. 4, Roosevelt Road, Taipei, 10617 Taiwan; 2 Center for Condensed Matter Sciences, National Taiwan University, No. 1, Sec. 4, Roosevelt Road, Taipei, 10617 Taiwan; 3 International Graduate Program of Molecular Science and Technology, National Taiwan University (NTU-MST), No. 1, Sec. 4, Roosevelt Road, Taipei, 10617 Taiwan; 4 Department of Chemical and Biomolecular Engineering, Sogang University, Baekbeom-ro 35, Mapo-gu, Seoul, 04107 Republic of Korea; 5 Center of Atomic Initiative for New Materials, National Taiwan University, No. 1, Sec. 4, Roosevelt Road, Taipei, 10617 Taiwan

**Keywords:** Structural properties, Carbon capture and storage, Metal-organic frameworks, Porous materials

## Abstract

Structural flexibility is a critical issue that limits the application of metal-organic framework (MOF) membranes for gas separation. Herein we propose a mixed-linker approach to suppress the structural flexibility of the CAU-10-based (CAU = Christian-Albrechts-University) membranes. Specifically, pure CAU-10-PDC membranes display high separation performance but at the same time are highly unstable for the separation of CO_2_/CH_4_. A partial substitution (30 mol.%) of the linker PDC with BDC significantly improves its stability. Such an approach also allows for decreasing the aperture size of MOFs. The optimized CAU-10-PDC-H (70/30) membrane possesses a high separation performance for CO_2_/CH_4_ (separation factor of 74.2 and CO_2_ permeability of 1,111.1 Barrer under 2 bar of feed pressure at 35°C). A combination of in situ characterization with X-ray diffraction (XRD) and diffuse reflectance infrared Fourier transform (DRIFT) spectroscopy, as well as periodic density functional theory (DFT) calculations, unveils the origin of the mixed-linker approach to enhancing the structural stability of the mixed-linker CAU-10-based membranes during the gas permeation tests.

## Abstract (web summary)

Metal–organic frameworks have demonstrated great promise for gas separation membranes, but their structural flexibility hinders their stability and hence sieving performance. Here, the authors systematically tune the ratio of mixed linkers in CAU-10-based MOF membranes, finding the optimal ratio that suppresses structural flexibility and enhances CO_2_/CH_4_ separation performance.

## Introduction

Metal-organic frameworks (MOFs) are an emerging class of crystalline microporous/mesoporous materials that have drawn increasing attention for various applications involving catalysis^1–3^, gas storage^4–6^, water harvesting^7–9^, and energy devices^10–12^. MOFs are considered promising candidates for membrane gas separations, which are energy-efficient processes that can be applied for the treatment of flue gas (separation of CO_2_/N_2_), the purification of natural gas (separation of CO_2_/CH_4_) or hydrocarbons (separation of olefin/paraffin). The previous studies suggest that a membrane with a selectivity of over 30 (for CO_2_/CH_4_ or CO_2_/N_2_) and a CO_2_ permeance of over 1000 GPU can reduce the cost of CO_2_ capture to 20–30 US$/ton^13^. MOFs can be processed in the form of mixed matrix membranes (MMMs) or pure MOF membranes for gas separations. Many MOF-based MMMs have shown good separation performance although they still need to be transformed to thin film composite membranes for industrial application. The MMM with Y-fum-fcu-MOF in 6FDA-DAM polyimide was reported to have a CO_2_/CH_4_ selectivity of approximately 30 with a CO_2_ permeability of nearly 1000 Barrer^14,15^. Hybrid UiO-66-CN@sPIM-1 membranes possessed a CO_2_/N_2_ selectivity of roughly 55 with a permeability of about 12,000 Barrer^16^. NUS-8 nanosheets/PIM-1 membranes with 2 wt% of the MOF exhibited a CO_2_/CH_4_ selectivity of approximately 30 and a CO_2_ permeability of nearly 6500 Barrer^17^. Hybrid Mg-MOF-74/PIM-1 membranes with 20 wt% of MOF showed a CO_2_/N_2_ selectivity of roughly 30 with CO_2_ permeability of about 11,000 Barrer^18^.

Different from MOF-based MMMs, relatively few pure MOF membranes showed good separation performance for CO_2_/N_2_ or CO_2_/CH_4_. IRMOF-1 membranes achieved CO_2_/N_2_ and CO_2_/CH_4_ selectivity of over 300 with a CO_2_ permeability of 10,000 Barrer^19^. MIL-160/CAU-10-F membranes have reached CO_2_/N_2_ and CO_2_/CH_4_ selectivities greater than 30 and 70, respectively. The CO_2_ permeability of the MIL-160/CAU-10-F membranes was approximately 2,000 Barrer^20^. ZIF-94 membranes showed a CO_2_/CH_4_ selectivity of nearly 38 with a CO_2_ permeability of about 28 Barrer^21^. In addition to the experimental studies in MOF-based membranes for CO_2_ separations^22–28^, computational works have also been conducted to identify promising materials for CO_2_ separation^29,30^ and to investigate the mechanism for CO_2_ transport within MOFs^31–33^.

In previous study, CAU-10-H, represent by the chemical formula [Al(OH)BDC)]·solvent (BDC = benzene-1,3-dicarboxylic acid), was first reported by Reinsch et al.^34^. The inorganic building unit (IBU) of CAU-10-H has helical AlO_6_ polyhedra chains that share *cis*-corner OH^-^ anions and bidentate carboxylate groups in their linker molecules. The structure of CAU-10-H comprised one-dimensional channels along *c*-axis, with a pore-limiting diameter (PLD) equaled to 3.2 Å, which is comparable to the kinetic diameter of CO_2_, N_2_, and CH_4_^35^. Recently, our group fabricated dense CAU-10-H membranes with a high CO_2_/CH_4_ selectivity (up to 50) and a CO_2_ permeability of 220 Barrer^35^. After that work, CAU-10-PDC, a derivative compound to CAU-10-H with BDC replaced by pyridine-3,5-dicarboxylic acid (PDC) as a linker, have been processed to a dense membrane for similar applications^36^. In addition, the structural similarity between CAU-10-H and CAU-10-PDC was verified through structure refinement, suggesting that both structures belong to the same space group^36^. The pure CAU-10-PDC membrane achieved a CO_2_/CH_4_ selectivity of over 60 with a CO_2_ permeability of over 20 Barrer. However, a severe structural deformation was found for the CAU-10-PDC membrane under exposure to CH_4_, which led to a pronounced change in the PLD from 4.15 to 2.95 Å.

The structural flexibility of MOFs significantly affects their applications to membrane gas separations. The rigid structure of ZIF-8 has a PLD of about 3.40 Å, and the pure ZIF-8 membranes had been expected to possess a high selectivity of CO_2_ (kinetic diameter, KD, of 3.30 Å) over N_2_ (KD of 3.64 Å) or CH_4_ (KD of 3.80 Å)^37^. Interestingly, most reported pure ZIF-8 membranes showed low selectivity for CO_2_/N_2_, but exhibited a very high selectivity of C_3_H_6_/C_3_H_8_ (over 300)^38–41^. This suggests that the effective aperture size of ZIF-8 is about 4.0–4.2 Å, instead of 3.40 Å. Follow-up studies suggest that those findings can be attributed to the linker rotation in ZIF-8, which enlarges the window size of the MOF for gas separations^37,42–48^. Recently, a rapid heat treatment (RHT) on the ZIF-8 membrane was developed to suppress the lattice flexibility^49^. Following the RHT, the pure ZIF-8 membrane can achieve a CO_2_/N_2_ selectivity of over 20. In different studies a mixed-linker approach has been used to suppress the gate-opening effects in ZIF-8^50,51^.

**Fig. 1:**
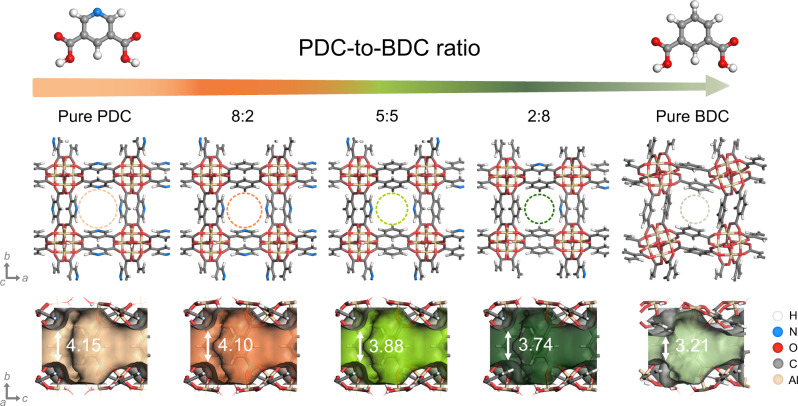
Mixed-linker CAU-10-PDC-H. Schematic illustrations of the mixed-linker approach to adjusting the crystal structure and the aperture size of the CAU-10 MOFs. The PLD decreases with the decrease of the PDC-to-BDC ratio. The dotted cycles in the middle row indicate PLDs of mixed-linker CAU-10-PDC-H. The values of PLDs (unit: Å) and the Connolly surface (calculated with a probe radius of 1.0 Å) are shown in isosurfaces in the bottom row.

Herein we propose a mixed-linker approach to suppress the structural flexibility of the CAU-10-based membranes, which can enhance the stability of the membranes during gas permeation tests. While the pure CAU-10-PDC membrane possesses good CO_2_/CH_4_ selectivity (over 60), it struggled with severe structural deformation under exposure to CH_4_. In this work, we partially replaced PDC with BDC to improve the lattice rigidity of the membrane. We synthesized mixed-linker CAU-10-PDC-H membranes with the mixed-ligand strategy in different PDC-to-BDC ratios, as shown in Fig. 1. The samples were subject to comprehensive characterization with nuclear magnetic resonance (NMR) spectroscopy, elemental analysis (EA), and scanning electron microscopy (SEM). We conducted single- and mixed-gas tests on the CAU-10-PDC-H membranes to evaluate their gas separation performance for CO_2_/N_2_ and CO_2_/CH_4_. X-ray diffraction (XRD) and diffuse reflectance infrared Fourier transform (DRIFT) spectroscopy were performed in situ under CO_2_ and CH_4_ to probe the structural deformation of the CAU-10-PDC-H. Computations based on the density functional theory (DFT) and energy decomposition analysis (EDA) were performed to support the experimental findings.

## Results

### CAU-10-PDC-H membranes

**Fig. 2:**
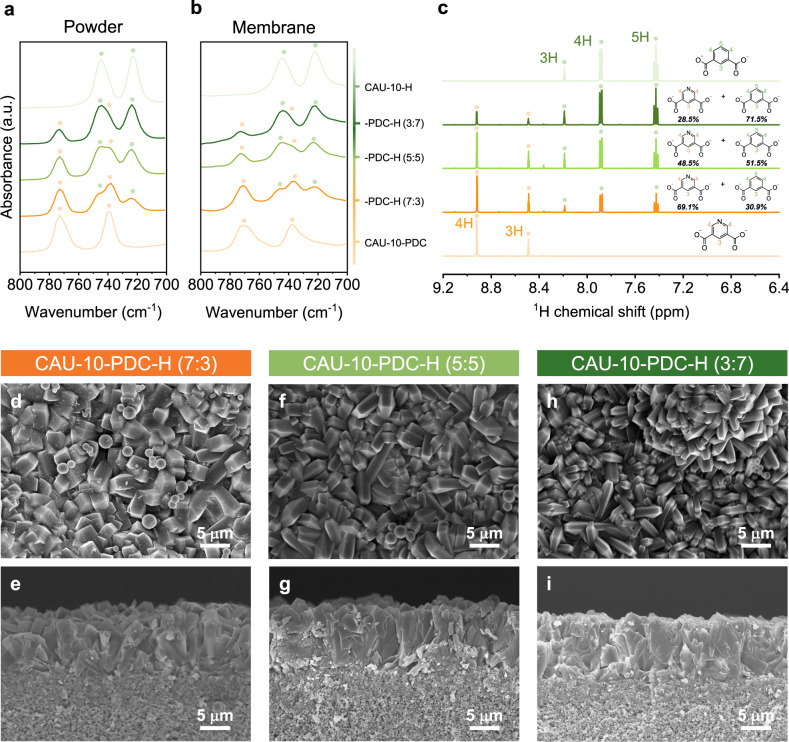
Composition and morphology of CAU-10-PDC-H powders and membranes. **a**, **b** FTIR spectra of CAU-10-PDC-H powders (**a**) and membranes (**b**) with various mixed-liker ratios, where the orange and green dots represent absorption from pure CAU-10-PDC and CAU-10-H, respectively. **c** ^1^H NMR spectra of the CAU-10-PDC-H powder samples, where the orange and tea green dots represent signals from PDC and BDC ligand, respectively. Light orange, orange, light green, dark green, and tea green lines represent CAU-10-PDC, CAU-10-PDC-H (7:3), (5:5), (3:7), and CAU-10-H, respectively. **d**–**i** Top-view (**d**, **f**, **h**) and cross-sectional (**e, g, i**) SEM images of CAU-10-PDC-H (7:3) (**d**, **e**), (5:5) (**f**, **g**), and (3:7) (**h**, **i**) membranes synthesized with the seeded growth method.

To prepare high-quality membranes, the seeded growth method was utilized for fabrication of CAU-10-PDC-H membranes on porous α-alumina substrate^52^. We then characterized the CAU-10-PDC-H samples in powder as well as in membrane form with FTIR spectroscopy. The FTIR spectra in the spectral range of 700–800 cm^−1^ are summarized in Fig. 2a, b, and those in a wider spectral range are presented in Supplementary Fig. 1. Two absorption peaks are observed for the CAU-10-H powder or membrane: 744 and 722 cm^−1^, which can be attributed to the 1,3-substituted benzene-rings (out-of-plane-deformation of C-H bonds)^34,53^. Absorption at 770 and 737 cm^−1^ is found for CAU-10-PDC powder or membrane, attributed to the 3,5 substituted pyridine-rings (out-of-plane-deformation of C-H bonds)^54^. For the powder or membrane samples formed with mixed linkers: CAU-10-PDC-H (3:7), CAU-10-PDC-H (5:5), and CAU-10-PDC-H (7:3), they have two sets of characteristic absorptions from the two parent materials. For CAU-10-PDC-H (3:7), the absorptions from CAU-10-H dominate the spectra; and those from CAU-10-PDC dominate the spectra of CAU-10-PDC-H (7:3). The results from FTIR analysis suggests the successful synthesis of the mixed-linker MOF membranes. To achieve a more quantitative analysis, we performed the liquid-state NMR characterization via dissolving powder samples dissolved in a mixed solvent formed from D_2_O and NaOH (Fig. 2c). The raw NMR spectra are present in Supplementary Figs. 2 and 3. The PDC molar percentage estimates via the NMR analysis for CAU-10-PDC-H (3:7), CAU-10-PDC-H (5:5), and CAU-10-PDC-H (7:3) are 28.5, 48.5, and 69.1%, respectively. The results from NMR agree with the stoichiometric ratio of the PDC versus BDC linker being used in the synthesis. Extensive quantifications for PDC-to-BDC ratios were also performed using FTIR and EA, and the results are summarized in Supplementary Table 1. The results obtained from different methods appear highly consistent.

The morphology of the powder and the membrane samples was characterized using SEM. The SEM images of the powder samples are shown in Supplementary Fig. 4. The crystal morphology of CAU-10-H, CAU-10-PDC, and the three CAU-10-PDC-H samples is poorly defined. The particle size of these powder samples is in a few micrometers with a broad size distribution. Despite the difference in chemical composition among these samples, we did not observe a clear difference in their micromorphology. Figure 2d–i and Supplementary Fig. 5 show the membrane samples’ top- and side-view SEM images. The membrane thicknesses of the CAU-10-PDC-H (3:7), CAU-10-PDC-H (5:5), and CAU-10-PDC-H (7:3) are approximately 10 μm. The pronounced intergrowth of MOF crystals is observed from the SEM images of the three samples, suggesting the high quality of the CAU-10-PDC-H membranes. The grain sizes of these three MOF membranes are subtly different. Specifically, the grain size reduces with the increase of the BDC linker in the MOF membranes. We revealed that the difference in grain size in mixed-linker membranes with varying ratios may be attributed to the different levels of deprotonation of the linkers. More precisely, the ligand of MOF must be deprotonated prior to forming metal-ligand bonds during MOF crystal growth^55^. Since PDC and BDC have different pKa values (2.8 and 3.7, respectively)^56^, PDC linkers have slightly higher dissociation than BDC linkers. This outcome could facilitate crystal growth due to the higher concentration of dissociated linker in the reaction solution.

The XRD patterns of the powder and the membrane samples are summarized in Supplementary Fig. 6a, b. While the XRD patterns from pure CAU-10-H and pure CAU-10-PDC are highly similar, the patterns from the three CAU-10-PDC-H samples do not present a considerable difference. However, a slight shift in the first peak, attributed to the (200) diffraction, indicates their difference in the pore size. Specifically, according to the structural analysis via the Rietveld refinement, the pure CAU-10-H has a PLD of 3.21 Å, and the PLD of pure CAU-10-PDC is 4.15 Å^35,36^. To learn the PLD of the mixed-linker CAU-10-PDC-H, we constructed three models in silico with different PDC-to-BDC ratios and ran the structural relaxation using the CASTEP module of the Materials Studio software. Due to the difficulty in constructing 7:3 and 3:7 models in CAU-10-PDC-H structures, our simulation included models with ratios of 8:2, 5:5, and 2:8. (Supplementary Fig. 7). The PLD gradually increases with the substitution of PDC because the configuration of PDC is smaller than BDC. Interestingly, the pore size distribution (PSD) is narrower when the substitution ratio of PDC increases. This suggests that CAU-10-PDC-H in a higher PDC ratio might have a more substantial sieving effect and higher permeability for gas mixtures. The N_2_ adsorption isotherms at 77 K and the derived BET surface areas are summarized in Supplementary Figs. 8a, b^57,58^. Among the three mixed-linker tested samples, CAU-10-PDC-H (7:3) possesses the highest BET surface area, which may render it a better solubility to CO_2_ and CH_4_ as compared to other ratios of mixed-linker samples.

### Gas separation performance of CAU-10-PDC-H membranes

**Fig. 3:**
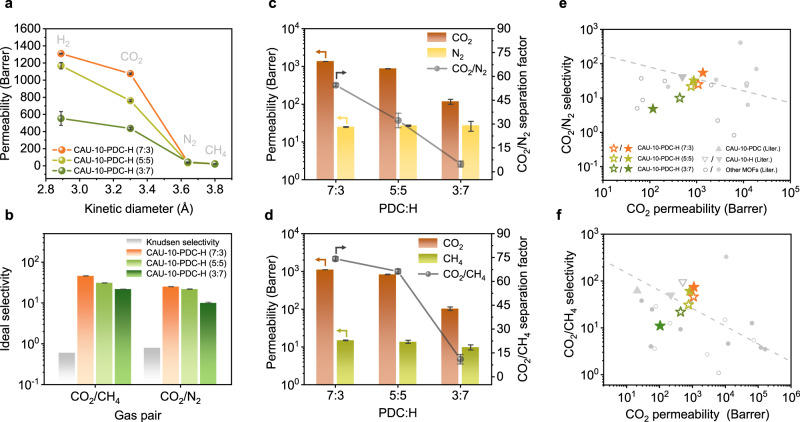
Gas permeation performance of CAU-10-PDC-H membranes. **a**, **b** Single-gas permeability (**a**) and ideal selectivity (**b**) of CO_2_/CH_4_ and CO_2_/N_2_ of CAU-10-PDC-H membranes under feed pressure of 2 bar at 35°C. **c**, **d** Mixed-gas permeability and separation factor of CAU-10-PDC-H membranes under feed pressure of 2 bar at 35°C for CO_2_/N_2_ (**c**) and CO_2_/CH_4_ (**d**), where the binary-gas feed is in a molar ratio of 50:50. **e**, **f** Robeson plots of selectivity versus permeability for separation performance of CAU-10-PDC-H membranes (stars) compared to literature data, including pure CAU-10-PDC (triangle), pure CAU-10-H (upside down triangles), and other MOF membranes (circles) when applied to CO_2_/N_2_ (**e**) and CO_2_/CH_4_ (**f**) gas pairs. We note that open and filled symbols respectively represent data from single- and mixed-gas measurements. Orange, light green, and dark green represent CAU-10-PDC-H (7:3), (5:5), and (3:7), respectively. Dashed lines indicate the performance upper bound for polymeric materials reported in 2008^92^. The raw data in **e** and **f** were summarized in Supplementary Table 6. The results presented in **a**–**f** represent the average performance of the three most effective membranes, based on a sample size of fewer than 10.

Single-gas permeation tests with H_2_, CO_2_, N_2_, or CH_4_ at 2 bar of partial pressure and 35°C were performed on the CAU-10-PDC-H membranes (Fig. 3a, b and Supplementary Tables 2 and 3). The permeation cutoff appears in between CO_2_ and N_2_, whose kinetic diameters are 3.30 and 3.64 Å, respectively. The CAU-10-PDC-H (7:3) membrane presents the highest permeability values of H_2_ and CO_2_, and the CAU-10-PDC-H (3:7) membrane has the lowest permeability for these two gases. This is consistent with the pore sizes of these three MOFs: CAU-10-PDC-H (7:3) > CAU-10-PDC-H (5:5) > CAU-10-PDC-H (3:7). The permeability coefficients of N_2_ and CH_4_ for the three membranes are considerably lower than their permeability estimates of CO_2_, leading to high ideal selectivity of CO_2_/N_2_ and CO_2_/CH_4_. The ideal selectivity of the three mixed-linker MOF membranes is distinct from the Knudsen selectivity, suggesting the gas permeation within these membranes is dominated by the ultramicropores of MOFs instead of pinholes from at the grain boundary in the membranes.

Mixed-gas permeation tests were also performed on the CAU-10-PDC-H membranes to examine their gas separation performance. The results are summarized in Fig. 3c, d and Supplementary Tables 4 and 5. These experiments were conducted with mixtures of CO_2_/CH_4_ and CO_2_/N_2_ at a molar ratio of 50:50 and 35°C. The total pressure at the feed side was set to be 2 bar. Similar to the single-gas permeation results, the CAU-10-PDC-H (7:3) membrane presents the highest separation factor for both mixtures. The CAU-10-PDC-H (3:7) membrane has the lowest separation performance in terms of CO_2_ permeability and the separation factors for the CO_2_/CH_4_ and CO_2_/N_2_ mixtures. While the three CAU-10-PDC-H membranes have very similar CH_4_ permeability, their separation performance is dominated by the permeation of CO_2_. Among the three samples, the CAU-10-PDC-H (7:3) membrane possesses the highest CO_2_ permeability, which can be attributed to its largest PLD and surface area. The high performance of the CAU-10-PDC-H (7:3) membrane in molecular sieving could also be attributed to its narrow PSD, which is discussed in the preceding section. Interestingly, we observed that the separation factor for CO_2_/N_2_ and CO_2_/CH_4_ is higher than the ideal selectivity. Furthermore, our mixed-gas measurements showed that the N_2_ and CH_4_ permeabilities were lower in comparison to their single-gas measurements. Moreover, we found that CH_4_ caused a slight decrease in CO_2_ permeability in the CO_2_/CH_4_ mixture, as compared to the CO_2_/N_2_ mixture. These results suggest that the competitive adsorption-diffusion between CO_2_ and other gases (N_2_ and CH_4_) leads to an increase in separation factor and a reduction in gas permeability in the mixed-gas measurements^19,59^.

The gas separation performance of the mixed-linker membranes is shown in the Robeson-type plots (Fig. 3e, f) and is compared to other existing MOF membranes, including pure CAU-10-H and pure CAU-10-PDC membranes^19,21,35,36,49,60–68^. The data presented in Fig. 3e, f are detailed in Supplementary Table 6. The CAU-10-PDC-H (7:3) and CAU-10-PDC-H (5:5) membranes outperform most existing MOF membranes in terms of CO_2_/N_2_ or CO_2_/CH_4_ separation. Interestingly, the gas separation performance of the mixed-linker CAU-10-PDC-H (7:3) membrane also exceeds their parent materials: pure CAU-10-H and pure CAU-10-PDC^35,36^. This could be attributed to its ideal pore dimensions, which maximize both selectivity and permeability. To ensure optimal separation performance of the CAU-10-PDC-H (7:3) membrane among different ratios of mixed-linker, we also fabricated CAU-10-PDC-H (8:2) membranes and examined their performance (Supplementary Fig. 9 and Supplementary Tables 4 and 5). Our results showed that the CAU-10-PDC-H (8:2) membrane exhibited a lower separation factor (<20) compared to the separation factors of CAU-10-PDC-H (7:3) and CAU-10-PDC-H (5:5), despite having a high permeability (>1000 Barrer). According to the abovementioned PLD of mixed-linker CAU-10-PDC-H, CAU-10-PDC (8:2) had a PLD of 4.10 Å, which is much larger than the kinetic diameters of CO_2_, N_2_, and CH_4_. That indicated that aforementioned gas molecules could easily penetrate through the MOF channels, resulting in a low separation performance.

Gas permeability is defined as a product of the sorption coefficient and the diffusion coefficient^69^. The sorption coefficient can be obtained by calculating the gas adsorption isotherms through dividing the adsorption uptake by a specific pressure. We conducted gas adsorption with CO_2_, N_2_, or CH_4_ on the CAU-10-PDC-H powder samples, and the resulting isotherms are summarized in Supplementary Fig. 10a–c. For the adsorption of CO_2_ and CH_4_, the three mixed-linker MOFs present almost identical adsorption quantity. As for the adsorption of N_2_, CAU-10-PDC-H (3:7) has slightly higher gas uptake than the other two samples. The adsorption quantities at 2 bar were used to derive the sorption coefficients, which were then used for obtaining the diffusion coefficients (Supplementary Fig. 10d, e). While the three mixed-linker MOFs present highly similar sorption coefficients for CO_2_, N_2_, or CH_4_, they have distinct diffusion coefficients. This finding suggests that the separation performance of the CAU-10-PDC-H membranes is dominated by gas diffusion. Specifically, CAU-10-PDC-H (7:3) possesses the highest diffusion coefficient of CO_2_, rendering it the most increased CO_2_ permeability and selectivity among the three mixed-linker MOF membranes studied herein.

## Discussion

**Fig. 4:**
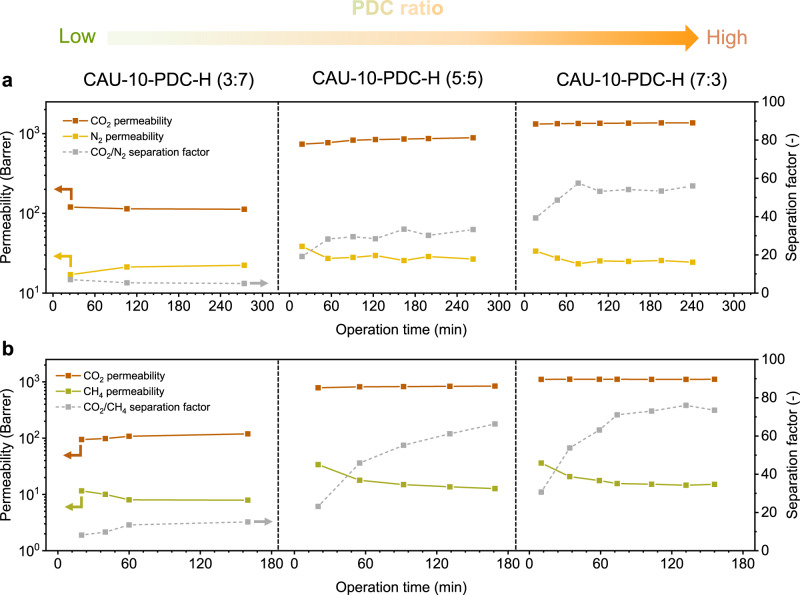
Long-term measurements of membrane gas separation. Permeability and separation factor of CAU-10-PDC-H membranes as a function of operation time from mixed-gas tests using **a** CO_2_/N_2_ and **b** CO_2_/CH_4_, respectively under feed pressure of 2 bar at 35 °C. Each mixture is in a molar ratio of 50:50.

In our previous study, we observed an ageing effect of the pure CAU-10-PDC membrane when it was exposed to CH_4_^36^. Specifically, when the CAU-10-PDC membrane was exposed to a mixture of CO_2_/CH_4_ (50/50) at a total pressure of 2 bar at 35°C, we observed a dramatic decrease in CH_4_ permeance over one order of magnitude in the period of 100 min. A change in space group, from *I*4_1_/*amd* to *I*4_1_, was also observed for CAU-10-PDC treated with CH_4_. Herein we assessed the stability of the mixed-linker CAU-10-PDC-H membranes for the operation of gas separation (Fig. 4). The CAU-10-PDC-H (7:3) membrane presents excellent stability for the separation of CO_2_/N_2_ as well as CO_2_/CH_4_. Specifically, this membrane’s gas permeability and selectivity barely change during the experiment. Interestingly, a slight decrease in the permeability of CH_4_, less than two times, is found for the membranes of CAU-10-PDC-H (5:5) and CAU-10-PDC-H (7:3), which is far less pronounced than the previous findings for the pure CAU-10-PDC membranes. The results suggest that incorporating the BDC linker into the framework of CAU-10-PDC makes this membrane more robust for gas separation.

**Fig. 5:**
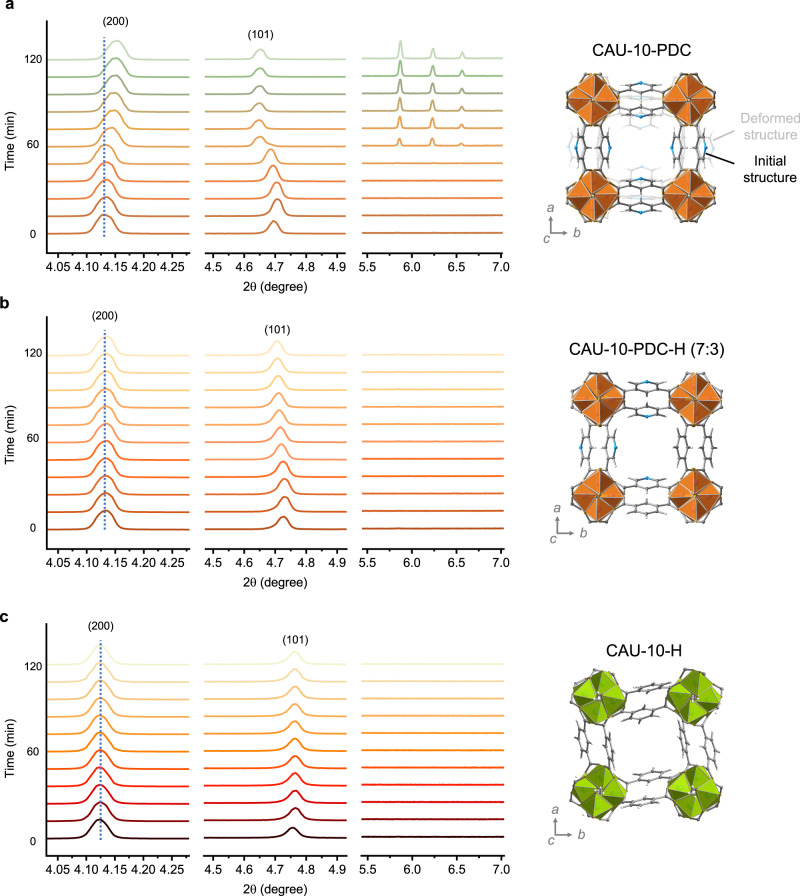
Structural deformation analysis. Time-resolved XRD patterns of **a** CAU-10-PDC, **b** CAU-10-PDC-H (7:3), and **c** CAU-10-H exposed to CH_4_ at 2 bar and 35°C obtained using a synchrotron X-ray source at 0.77489 Å. The dotted lines indicate the peak shift of (200) diffraction, which appears for CAU-10-PDC but is absent for CAU-10-H and CAU-10-PDC-H (7:3).

Time-resolved powder XRD was performed further to probe the structural stability of the mixed-linker MOFs. The XRD measurements were conducted in situ under 2 bar of CH_4_ at 35 °C (Fig. 5 and Supplementary Fig. 11). The three mixed-linker MOFs do not present a noticeable peak shift for their (200) or (101) diffraction when exposed to CH_4_ for 2 h. This suggests good stability of these mixed-linker MOFs when exposed to CH_4_. However, the (200) diffractions from the mixed-linker MOFs show a slight reduction in intensity, which can be attributed to the adsorption of CH_4_. Unlike the mixed-linker MOFs, pure CAU-10-PDC presents a pronounced peak shift for the (200) diffraction, corresponding to a dramatic change in the aperture size for gas transport. Specifically, the PLD of CAU-10-PDC reduces from 4.15 to 2.95 Å after the treatment with CH_4_, making it nearly impermeable to gases^36^. The deformed structure of CAU-10-PDC was determined via the Rietveld refinement and is shown in Fig. 5. The results also indicate that partial replacement of PDC with BDC can significantly improve the lattice rigidity under methane.

**Fig. 6:**
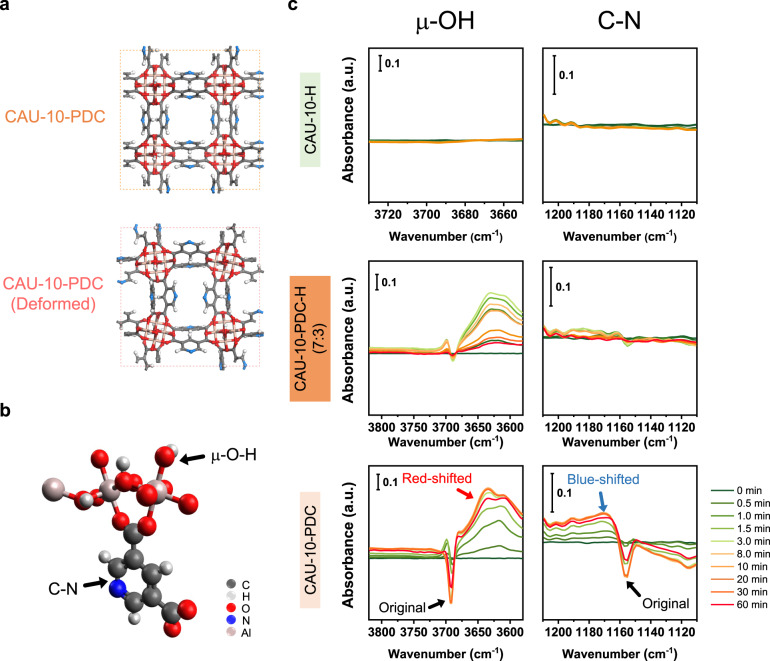
In situ DRIFT experiments for the CAU-10 MOFs. **a** CAU-10-PDC models used for the DFT calculations for the IR absorbance. The results are shown in Supplementary Table 7, where peak shifts are found for the μ-OH and the C-N bonds between the two structures. **b** Illustration of the CAU-10-PDC structure, where the arrows indicate the μ-OH and the C-N bonds. **c** DRIFT spectra of the CAU-10-H, CAU-10-PDC-H (7:3), and CAU-10-PDC membranes under exposure to CH_4_. In the DRIFT spectra of CAU-10-PDC, red and blue shifts are found for the μ-OH and the C-N bonds, respectively. These shifts are absent in the spectra of CAU-10-H. In the spectra of CAU-10-PDC-H (7:3), the blue shift of the C-N bond is absent, and the red shift of the μ-OH reaches the maximum intensity in the first 3 min and gradually decrease until 60 min.

To gain insight into the gas adsorption-induced structural change, we performed DRIFT spectroscopy on the CAU-10-based membranes being exposed to CH_4_ (Supplementary Fig. 12). The recorded absorbance in these spectra is relative to the one at time = 0 when the system is fully evacuated. Both upward and downward peaks appear following the introduction of CH_4_ to the system. The upward peaks represent the new absorbance after gas adsorption, whereas the downward ones represent the reduction in the intensity of the existing absorbance. No upward or downward peaks appear in the spectra of the CAU-10-H membrane, suggesting its structure is unaffected by CH_4_. On the other hand, dramatic changes are found for the CAU-10-PDC sample. Specifically, absorbance corresponding to the μ-OH and C-N groups varies. The μ-OH group is associated with the aluminum metal cluster^70^. In contrast, the C-N group is associated with the PDC linker (Fig. 6a, b)^71^. Upon the adsorption of CH_4_, the μ-OH group from the CAU-10-PDC membrane shows a significant red shift of almost 100 cm^−1^, when the C-N group presents a blue shift (Fig. 6c). Both of the peak changes are associated with remarkable peak broadenings. The peak shift suggests that the CH_4_ adsorption could initiate a structural change within the CAU-10-PDC membrane, and the peak broadening indicates multiple modes of such a change. Such a peak shift could be caused by the interaction between the adsorbate molecules and the PDC linker, which alters the bond lengths of the linkers and the μ-OH group from the metal cluster. The DFT simulations were applied to calculate the FTIR vibrational frequencies of the as-made and the deformed CAU-10-PDC structures (Fig. 6a), which were determined by the XRD-based analysis. The DFT calculations suggest that the structure deformation can cause a significant red shift of the μ-OH group and a blue shift of the C-N group (Supplementary Table 7).

Interestingly, the peak shift of μ-OH in the CAU-10-PDC-H (7:3) membrane seems temporal and less severe than that in the CAU-10-PDC analog. Specifically, the red shift of the μ-OH group reached a maximum in the first three minutes and then gradually weakened. At the end of the measurement (60 min), the red shift of the μ-OH group was barely observed. In addition, nearly no peak shift of C-N was found in the CAU-10-PDC-H (7:3) membrane. The results suggest that adding 30% of BDC to the CAU-10-PDC membrane can considerably stabilize the structure under exposure to CH_4_. The DRIFT spectral study agrees with the XRD characterization and membrane gas permeation results. We also applied the in situ DRIFT spectroscopy analysis under the adsorption of CO_2_ (Supplementary Fig. 13). Similar peak shifts appear for the functional groups of μ-OH and C-N in the membrane samples following the CO_2_ exposure. Nevertheless, the peak shifts under CO_2_ exposure are much less significant than the CH_4_ one. In addition, the grain boundary microstructure of the CAU-10-PDC-H (7:3) membrane may be well maintained under exposure to either CO_2_ or CH_4_ or their mixtures. The peak shifts observed from the aforementioned DRIFT spectra are summarized in Supplementary Table 8.

To explain how the structural flexibility of CAU-10-PDC is suppressed via mixed-linker approach, we employed EDA to investigate the methane adsorption behavior of CAU-10-PDC and CAU-10-H (Supplementary Fig. 14). Our EDA results indicate that the binding energy of CAU-10-PDC with the induced deformation was calculated to be −2.58 kcal mol^−1^, indicating that the host-guest interaction can cause a deformation in the structure to reach a lower energy state when exposed to methane. In contrast, when exposed to methane, the binding energy of CAU-10-H with the induced deformation is positive (1.69 kcal mol^−1^), suggesting that it is less likely to undergo structural deformation. Based on the EDA, we conclude that the PDC linker exhibits a stronger interaction energy (−11.7 kcal mol^−1^) with methane than the BDC linker (−6.45 kcal mol^−1^). Therefore, adsorption of methane in CAU-10-PDC releases more energy, which may cause structural change (this change only needs 9.12 kcal mol^−1^). However, by replacing a portion of the PDC linkers with BDC linkers, we can reduce the probability of framework deformation by decreasing the energy released during host-guest adsorption

In summary, we studied the origin of the structural deformation of MOF membranes under exposure to gases. Pure CAU-10-PDC membrane has a high CO_2_/CH_4_ separation factor (62). However, the strong interaction between the PDC linker and the adsorbate molecules (CO_2_ or CH_4_) leads to a significant structural change in CAU-10-PDC. The structural deformation was investigated by the in situ characterizations with XRD and DRIFT spectroscopy. The mixed linker approach studied in this work, via mixing PDC and BDC linkers in a MOF, could engineer the aperture size and the gas adsorption properties of the CAU-10-based MOFs and suppress the flexibility observed in CAU-10-PDC. Specifically, a 30 mol.% substitution of PDC with BDC can significantly stabilize the MOF structure during the gas adsorption and permeation. For the optimized CAU-10-PDC-H (70/30), the structural change is barely observed in the in situ analysis with XRD or DRIFT spectroscopy. The CAU-10-PDC-H (70/30) membrane presents a CO_2_/CH_4_ separation factor of 74.2 and a CO_2_ permeability of 1,111.1 Barrer under 2 bar of feed pressure at 35°C.

## Methods

### Chemicals and materials

Al_2_(SO_4_)_3_·18H_2_O was purchased from JT Baker. Pyridine-3,5-dicarboxylic acid (3,5-H_2_PDC, 98%) and benzene-1,3-dicarboxylic acid (1,3-H_2_BDC, 99%) were purchased from Alfa Aesar. Hereafter 3,5-H_2_PDC and 1,3-H_2_BDC were respectively referred to as PDC and BDC. *N,N*-dimethylformamide (DMF, 99.8%) and methanol (MeOH, 99%) were purchased from Macron. All chemicals were used without further purification. The deionized water (DI water) used for synthesis was purified using an ELGA VEOLIA PURELAB® classic analytical ultrapure water system. Porous *α*-alumina substrates were purchased from the CHAO YUE Diamonds Ltd. Co. The substrates were composed of *α*-alumina particles with an average particle size of approximately 400 nm, and they possessed a diameter of 40 mm, a thickness of 2 mm, and a porosity of 34%.

### Synthesis of CAU-10-PDC-H powder

For the synthesis of CAU-10-PDC, PDC (5 mmol) was added to DMF (6 ml). The mixture was sonicated using an ultrasonication bath until the solid content was completely dissolved. A separate solution was prepared by dissolving Al_2_(SO_4_)_3_·18H_2_O (5 mmol) in DI water (24 ml). The two solutions mentioned above were then mixed and refluxed under agitation at 120 °C for 2 days. MOF particles formed during the reaction. The solvent was removed from the suspension via vacuum filtration. The solid product was dispersed in methanol and agitated at room temperature for 1 day, which allowed for the removal of DMF or water as guest molecules in the MOF. Vacuum filtration was applied for the removal of methanol. The powder sample was dried in a convection oven at 100 °C for 1 day.

The synthesis of CAU-10-H resembled that of CAU-10-PDC, except that PDC (5 mmol) was replaced by BDC (5 mmol). The synthesis of mixed-linker CAU-10-PDC-H (7:3) also resembled the synthesis of pure CAU-10-PDC, except that PDC (3.5 mmol) and BDC (1.5 mmol) were used in the first step. The synthesis of mixed-linker CAU-10-PDC-H (5:5) or CAU-10-PDC-H (3:7) was conducted in the aforementioned manner; and the quantity of PDC and BDC used in the synthesis was respectively (2.5 and 2.5 mmol) or (1.5 and 3.5 mmol).

### Deposition of CAU-10-PDC-H seed layer

Prior to deposition, the *α*-alumina substrate was immersed in DI water and cleaned using an ultrasonication bath for 24 h and then dried in a convection oven at 105 °C for at least 24 h. The powder of CAU-10-PDC, CAU-10-H, or mixed-linker CAU-10-PDC-H was dispersed in DI water to form a 0.2 wt% suspension. Approximately 2.5 ml of the suspension was applied dropwise onto the substrate followed by spin-on deposition at 2000 rpm for 30 s. The spin-on deposition was performed using a Laurell spin coater (Model-WS-650M2-23NPPB). The sample was placed in a convection oven at 100 °C for 20 min. The deposition procedure mentioned above was repeated for two more times on the same substrate to increase the coverage of the MOF seed layer.

### Secondary growth of CAU-10-PDC-H membrane

The *α*-alumina substrate deposited with a seed layer was placed in a Teflon-lined autoclave with a maximum capacity of 200 ml. The substrate was mounted in a proprietary Teflon holder where the seed layer faced up (Supplementary Fig. 15). A solution composed of Al_2_(SO_4_)_3_·18H_2_O (0.83 mmol), PDC (*x* mmol), BDC (*y* mmol), DI water (32 ml), and DMF (8 ml) was added to the Teflon-lined autoclave for the secondary growth of CAU-10-PDC-H membrane, wherein (*x*, *y*) was (0.83, 0), (0.581, 0.249), (0.415, 0.415), (0.249, 0.581), or (0, 0.83) respectively for the synthesis of CAU-10-PDC, CAU-10-PDC-H (7:3), CAU-10-PDC-H (5:5), CAU-10-PDC-H (3:7), or CAU-10-H membrane. The autoclave was heated in a convection oven at 100 °C for 24 h for the secondary growth of a dense MOF membrane. Following the secondary growth, the membrane sample was immersed in methanol (100 ml) under agitation at room temperature for one day for the removal of DMF. The sample was dried at 100°C for 1 day prior to use.

### Materials characterization

An in-house X-ray diffractometer, Rigaku SmartLab SE, with Cu K*α* radiation was used for characterization of MOF powder as well as MOF membrane samples. During the measurements, the diffractometer was operated at 40 kV and 40 mA. XRD patterns for the powder samples were collected from 5 to 20° 2*θ* with a step size of 0.02° 2*θ* at a scanning rate of 2° min^−1^. The membrane samples were measured in grazing incidence mode using an incident beam fixed at 0.5°.

Time-resolved XRD was performed at Taiwan Photon Source (TPS) 19 A station at the National Synchrotron Radiation Research Center (NSRRC). The X-ray at a wavelength of 0.77489 Å (16 keV) was generated from a cryogenic undulator under vacuum (CU15). The proprietary setup for the measurements can be found in our previous report^36^. The CAU-10-PDC-H powder samples were packed in a capillary tube with a diameter of 0.7 mm. Prior to the measurement, the powder sample underwent degassing at 0.005 bar at 70 °C for at least 30 min. The sample was then cooled to 35 °C. CH_4_ at 2 bar was introduced to the capillary tube with the MOF sample. XRD patterns were continuously recorded at intervals of 12 min over a time span of up to 120 min. Each diffraction pattern was recorded using a MYTHEN 18 K position-sensitive detector with exposure duration of 1 s.

Fourier transform infrared (FTIR) spectra of MOF powder and membrane samples were acquired using a BRUKER ALPHA II FTIR spectrometer equipped with a KBr beam splitter. The measurements were conducted in the mode of attenuated total reflectance with a diamond crystal. Each spectrum was recorded from 120 scans at a spectral resolution of 4 cm^−1^. The PDC-to-BDC ratios of the CAU-10-PDC-H powder and membrane samples were derived from the peak areas at 770 cm^−1^ and 722 cm^−1^ for PDC and BDC, respectively.

In situ diffuse reflectance infrared Fourier transform (DRIFT) spectra of the MOF membranes were recorded by Bruker Tensor 27 FT-IR spectrometer with a HgCdTe detector for CO_2_ or CH_4_ adsorption process. All spectra were obtained with 32 scans and a spectral resolution 4 cm^−1^. Each FTIR spectrum took approximately 15 s. Supplementary Fig. 16 shows the cell configuration for the in situ DRIFT measurement. To study the effect of CO_2_ and CH_4_ adsorption process on the structure stability of MOF samples, the MOF membranes were treated by the sequential CH_4_ and CO_2_ adsorption process. First, a membrane sample was vacuumed in the reactor overnight to remove gases that were physically adsorbed in the sample. Then CH_4_ was purged into the chamber for the measurement. After that, the chamber was vacuumed for 1 h prior to the CO_2_ adsorption process.

^1^H NMR spectra were acquired using a Bruker AVIII 500 MHz NMR equipped with a cryo prodigy broadband probe. A missed solvent with 600 μl of D_2_O and 10 μl of 40 wt% NaOD in D_2_O was used for dissolving the powder sample in order to form a homogenous liquid for the measurement.

Elemental analysis (EA) of CHN was performed using an Elementar vario EL cube analyzer (for CHNS). First, samples were placed into a tin capsule and then in the autosampler to inject a high-temperature furnace. Secondly, samples were burned in the oxygen flow at temperature of up to 1800 °C. And then, the gases (N_2_, N_x_O_y_, CO_2_, H_2_O, SO_2_, and SO_3_) formed during the combustion were passed through a reduction tube to produce a gas mixture (N_2_, H_2_O, CO_2_, and SO_2_). The gas mixture was then passed through the adsorption column to separate the different gases. Finally, the gas composition was analyzed using a gas chromatograph equipped with a thermal conductivity detector. Note that N_2_ bypassed the adsorption column and was detected directly. The analysis was used acetanilide standard in the CHN module with <0.1% abs. for each element. Considering the composition of CAU-10-PDC-H, we set the molecular formula as [Al(OH)(BDC)_*x*_(PDC)_(1-*x*)_]·*y*H_2_O·*z*DMF, for the measurements.

A Hitachi S4800 field emission scanning electron microscope (SEM) was used to characterize the morphology of the membrane samples. All samples were placed in vacuum desiccator overnight to remove moisture. Prior to imaging, the samples were coated with platinum via sputtering deposition under acceleration voltage of 25 V for 40 s. SEM was performed under an acceleration voltage of 10 kV during image acquisition.

Gas adsorption isotherms of CO_2_, N_2_ and CH_4_ were measured at 35°C with the pressure decay method using a homemade device^72^. CAU-10-PDC-H powder samples were inserted into a Swagelok® filter element kit and wrapped loosely with aluminum foil. They were loaded into the sample chamber and degassed at 35 °C overnight before each measurement to remove any gases trapped in the sample^73^. The adsorption isotherms of all the samples were fitted by Langmuir model.

Nitrogen adsorption isotherms were obtained using a Micromeritics (ASAP2020) at 77 K. Before the measurement, approximately 0.1–0.3 g of powder sample was placed in a tube and degassed under 0.005 mbar at 160°C overnight. Combination of the excess sorption work (ESW) and the Brunauer–Emmett–Teller (BET) method was utilized to receive a more accurate surface area of the material^57,58^.

### Membrane gas permeation tests

The single-gas permeation test for the membrane samples were performed using a proprietary system based upon the constant-volume method^74^ (Supplementary Fig. 17). A membrane sample was placed in a cell sealed with aluminum tape and epoxy (3M^TM^ Scotch-Weld^TM^ Epoxy Adhesive DP100FR), and was outgassed at roughly 50 mtorr at room temperature for 12 h. After outgassing, the temperature of the system was set to be 35 °C. The system was then disconnected to the vacuum pump, and a target gas (H_2_, CO_2_, N_2_ or CH_4_) at a partial pressure of 2 bar was introduced the feed side. The pressure on the product side of the membrane started increasing due to the permeation of the target gas from the feed side, and it was monitored using an MKS AA09A Baratron transducer.

The increase of the downstream pressure as a function of time was then converted into the gas permeability for the membrane using the following equation:

1 $${{{{{{\rm{Permeability}}}}}}}=\frac{V}{{RTA}}\left(\frac{{{{{{{\rm{d}}}}}}p}}{{{{{{{\rm{d}}}}}}t}}\right)\frac{l}{\triangle p}$$

where *R* is the gas constant, *T* is temperature, *A* is the membrane area that allows for permeation, *V* is the volume of the downstream reservoir, $\frac{{{{{{{\rm{d}}}}}}p}}{{{{{{{\rm{d}}}}}}t}}$ is the pressure on the permeate side as a function of time, *l* is the thickness of membrane and ∆*p* is the transmembrane pressure difference. The ideal selectivity of one gas species over another was calculated as the ratio of the permeability of these two species obtained from their single-gas permeation tests. The effective membrane area used in for Eq. (1) was measured by a photographic image of the membrane sample with the aid of an open-source package, Image J^75^.

The same setup was used for the mixed-gas permeation tests, and these tests were conducted in a very similar manner to the single-gas permeation. The feed gas was composed of either CO_2_/N_2_ (50/50 in mol) or CO_2_/CH_4_ (50/50 in mol) at a total pressure of 2 bar at 35 °C. The gas composition on the product side was analyzed using a gas chromatograph (Shimadzu GC-2014) equipped with a thermal conductivity detector (TCD) and a Shincarbon-ST column. The separation factor of species *i* over *j* was computed using the following equation:

2 $${{{{{{\rm{Separation}}}}}}}\;{{{{{{\rm{factor}}}}}}}=\frac{{y}_{i}/{y}_{j}}{{x}_{i}/{x}_{j}}$$

where *x*_*i*_ and *y*_*i*_ are the molar fraction of *i* on the feed and on the permeate side, respectively; and *x*_*j*_ and *y*_*j*_ are the molar fraction of *j* on the feed and on the permeate side, respectively

### Computational methods

Structure relaxation of CAU-10-PDC-H structures were implemented using CASTEP module of Materials Studio suite^76^. The calculation was used in reciprocal space as pseudopotential representation. The exchange-correlations were applied using Perdew–Burke–Ernzerhof (PBE) functional in the generalized gradient approximation (GGA) of the plane wave pseudopotential method^77^. Ultrasoft pseudopotential was conducted to calculate the interactions between the ionic nucleus and valence electrons^78^. Broyden–Fletcher–Goldfarb–Shanno algorithm was utilized for geometry optimization^79^. The value of cut-off energy was set at 340 eV.

PLD and PSD of CAU-10-PDC-H were both estimated by using an open-source package, Zeo + +^80^, based on the CIF files corresponding to the optimized structures. Note that PSDs were implemented using a total of 50,000 Monte Carlo (MC) samples per unit cell with a probe radius of 1.1 Å^81^.

In the vibrational frequency analysis, the crystallographic structures of CAU-10-PDC and deformed CAU-10-PDC were used to determine the positions of all the atoms in the MOF models. Periodic density functional theory (DFT) calculations were performed using the PBE exchange-correlation functional within the generalized gradient approach (GGA)^77^, as implemented in VASP 5.4.4^82^. The valence density was expanded in a plane wave basis set with a kinetic energy cutoff 450 eV, where the effect of core electrons on valence density was considered using the projector-augmented wave method (PAW)^83^. A 2 × 2 × 4 Monkhorst–Pack k point mesh^84^ was used for integration over the Brillouin zone in reciprocal space for both geometry optimization and frequency calculations.

For the energy decomposition analysis (EDA)^85^, the PBE exchange-correlation functional^77^ with semiempirical dispersion corrections from the DFT-D3^86^ method was used for geometry optimizations and single point calculations, as implemented in the Quickstep module of CP2K package^87^. Double-ζ valence plus polarization MOLOPT basis set^88–91^, in conjunction with the relativistic, norm-conserving Goedecker–Teter–Hutter pseudopotentials, was used for all atoms. The auxiliary plane-wave basis set was defined by an energy cutoff of 450 Ry, accompanied by the relative cutoff of 60 Ry for the Gaussian basis set collocation. The binding energy (${E}_{{{{{{{\rm{bind}}}}}}}}$) between the guest molecule and the MOF system can be defined as:

3 $${E}_{{{{{{{\rm{bind}}}}}}}}=E\left({H\bullet G}_{{{{{{{\rm{def}}}}}}}}\right)-E\left(H\right)-E\left(G\right)$$

where $E\left({H\bullet G}_{{{{{{{\rm{def}}}}}}}}\right)$ corresponds to the energy of the deformed MOF-guest complex, and $E\left(H\right)$ and $E\left(G\right)$ represent the energies of the original MOF and guest molecule, respectively. In order to obtain a comprehensive understanding of the conformational change, an EDA was performed. Within the EDA framework, the $E\left({H\bullet G}_{{{{{{{\rm{def}}}}}}}}\right)$ can be further decomposed as:

4 $$E\left({H\bullet G}_{{{{{{{\rm{def}}}}}}}}\right)=E\left(H\right)+E\left(G\right)+{E}_{{{{{{{\rm{def}}}}}}}}+{E}_{{{{{\mathrm{int}}}}}}$$

where the deformation energy (${E}_{{{{{{{\rm{def}}}}}}}}$) refers to the energy that is required to distort the isolated MOF and guest molecule from their original geometries to the geometries they have in the deformed MOF–guest complex, and the interaction energy (${E}_{{{{{\mathrm{int}}}}}}$) represents the energy difference induced by the guest–host interactions. By substituting Eq. (3) into Eq. (4), we can express the binding energy as the sum of ${E}_{{{{{{{\rm{def}}}}}}}}$ and ${E}_{{{{{\mathrm{int}}}}}}$.

5 $${E}_{{{{{{{\rm{bind}}}}}}}}={E}_{{{{{{{\rm{def}}}}}}}}+{E}_{{{{{\mathrm{int}}}}}}$$

## Supplementary information

Supplementary Material

## Data Availability

Any relevant data are available from the corresponding authors upon reasonable request.
