# Supplementary material for: Mixed-linker strategy for suppressing structural flexibility of metal-organic framework membranes for gas separation
Source: Commun Chem. 2023 Jun 10;6:118. doi: 10.1038/s42004-023-00917-2 (PMC10257696; doi:10.1038/s42004-023-00917-2)
Supplement: Supplementary file 1 — Supplementary Material [file 42004_2023_917_MOESM1_ESM.pdf]

Supplementary Information

for

Mixed-linker strategy for suppressing structural flexibility of metal-organic framework membranes for gas separation

Chung-Kai Chang[a], Ting-Rong Ko[b], Tsai-Yu Lin[b, c], Yen-Chun Lin[a], Hyun Jung Yu[d], Jong Suk Lee[d,]*, Yi-Pei Li[a,]*, Heng-Liang Wu [b, e,]*, and Dun-Yen Kang[a,c,e]*

[a]*Department of Chemical Engineering, National Taiwan University, No. 1, Sec. 4, Roosevelt Road, Taipei 10617, Taiwan*

[b]*Center for Condensed Matter Sciences, National Taiwan University, No. 1, Sec. 4, Roosevelt Road, Taipei 10617, Taiwan*

[c]*International Graduate Program of Molecular Science and Technology, National Taiwan University (NTU-MST), No. 1, Sec. 4, Roosevelt Road, Taipei 10617, Taiwan*

[d]*Department of Chemical and Biomolecular Engineering, Sogang University, Baekbeom-ro 35, Mapo-gu, Seoul 04107, Republic of Korea*

[e]*Center of Atomic Initiative for New Materials, National Taiwan University, No. 1, Sec. 4, Roosevelt Road, Taipei 10617, Taiwan*

Jong Suk Lee *E-mail: jongslee@sogang.ac.kr

Yi-Pei Li *E-mail: yipeili@ntu.edu.tw

Heng-Liang Wu *E-mail: hengliangwu@ntu.edu.tw

Dun-Yen Kang *E-mail: dunyen@ntu.edu.tw

Supplementary Figures

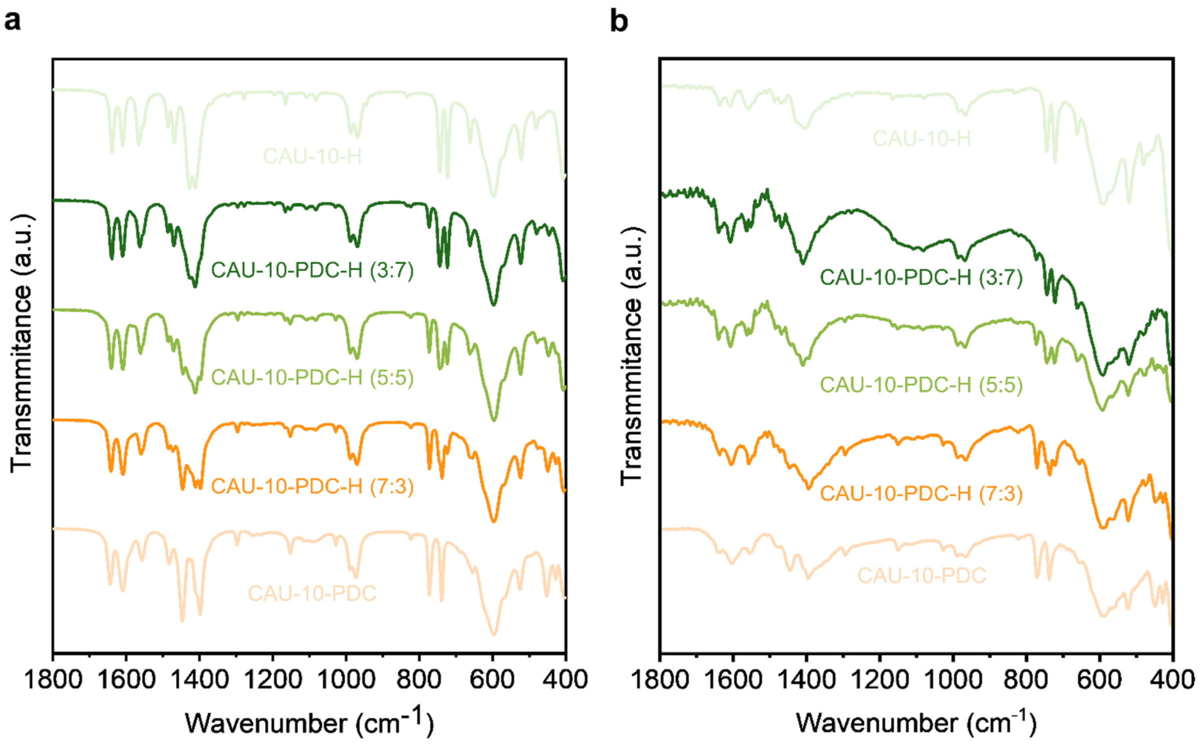

Supplementary Fig. 1. FT-IR spectra of CAU-10-PDC-H **a** powders and **b** membranes.

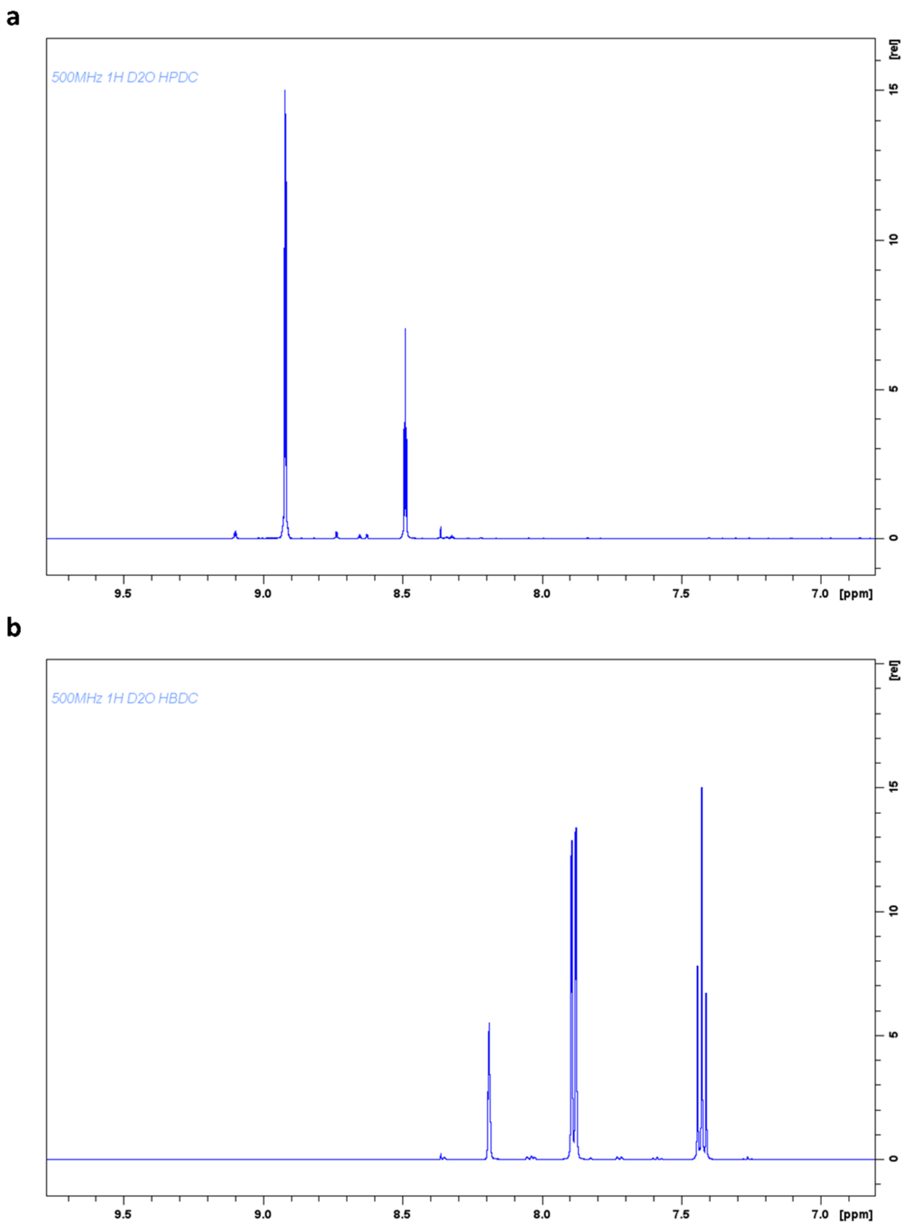

Supplementary Fig. 2. ^{1}H NMR spectra of **a** CAU-10-PDC and **b** CAU-10-H powder samples.

a

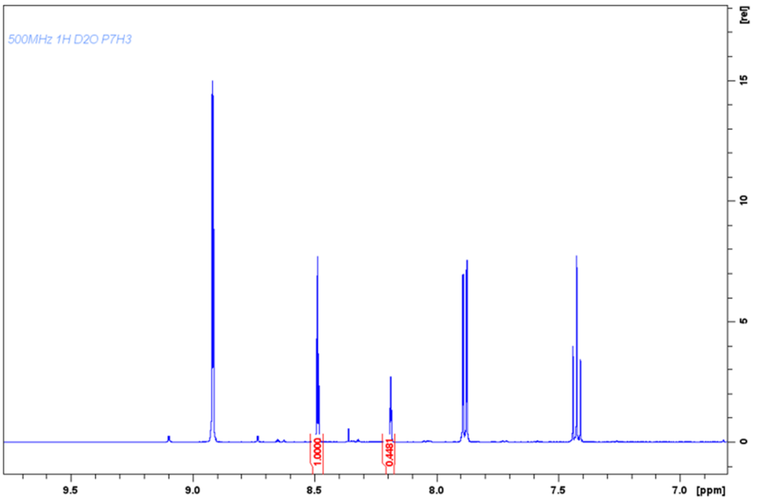

b

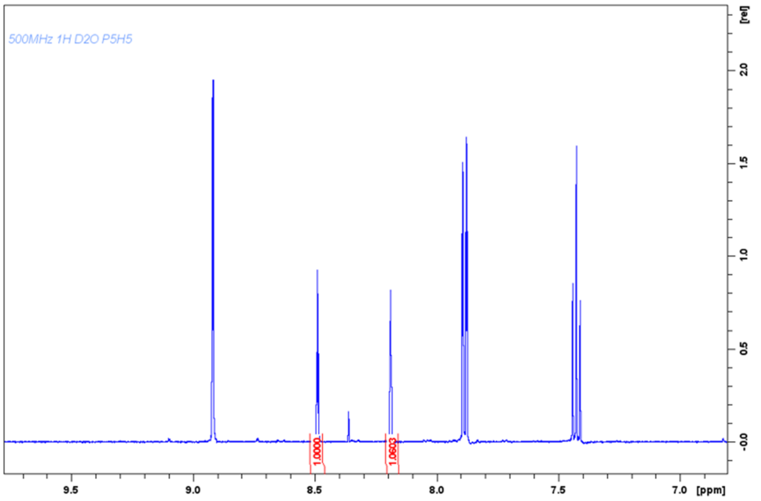

c

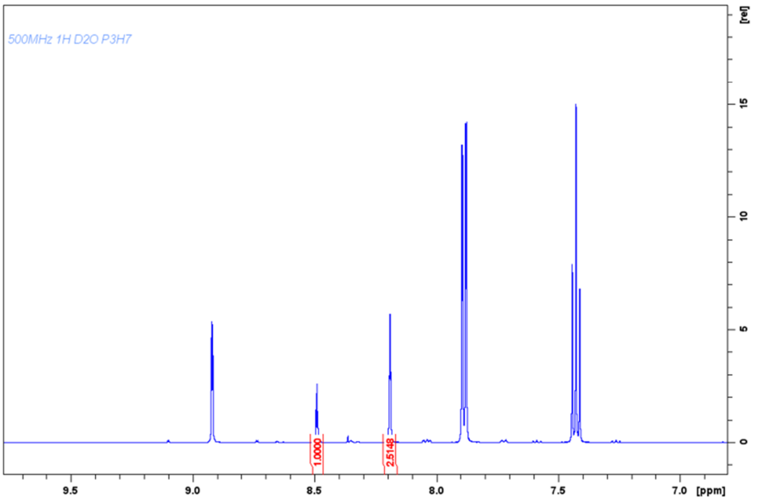

Supplementary Fig. 3. ^{1}H NMR spectra of **a** CAU-10-PDC-H (7:3), **b** (5:5), and **c** (3:7) powder samples.

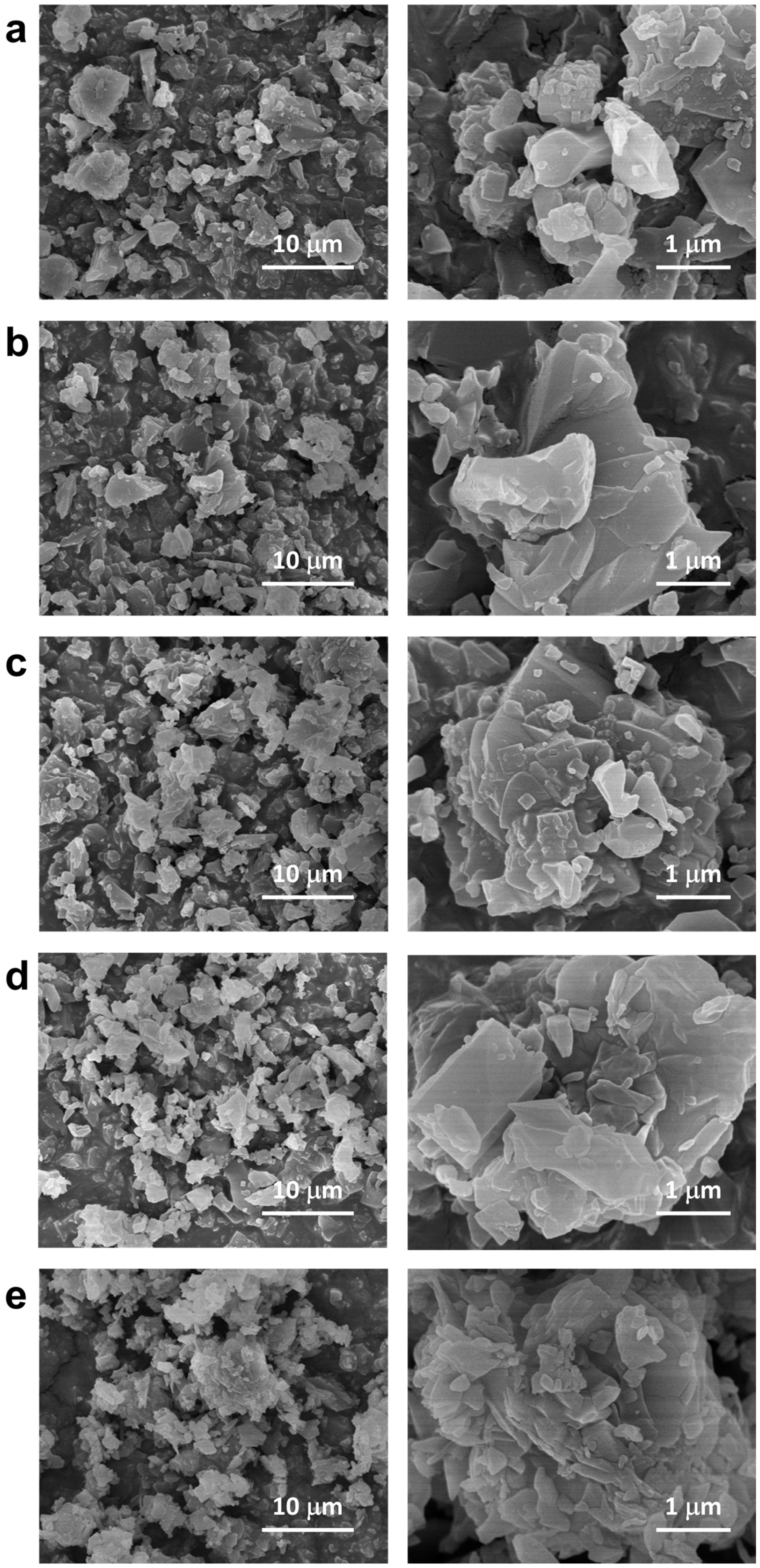

Supplementary Fig. 4. SEM images of **a** CAU-10-PDC, **b** CAU-10-PDC-H (7:3), **c** CAU-10-PDC-H (5:5), **d** CAU-10-PDC-H (3:7), and **e** CAU-10- H powder samples.

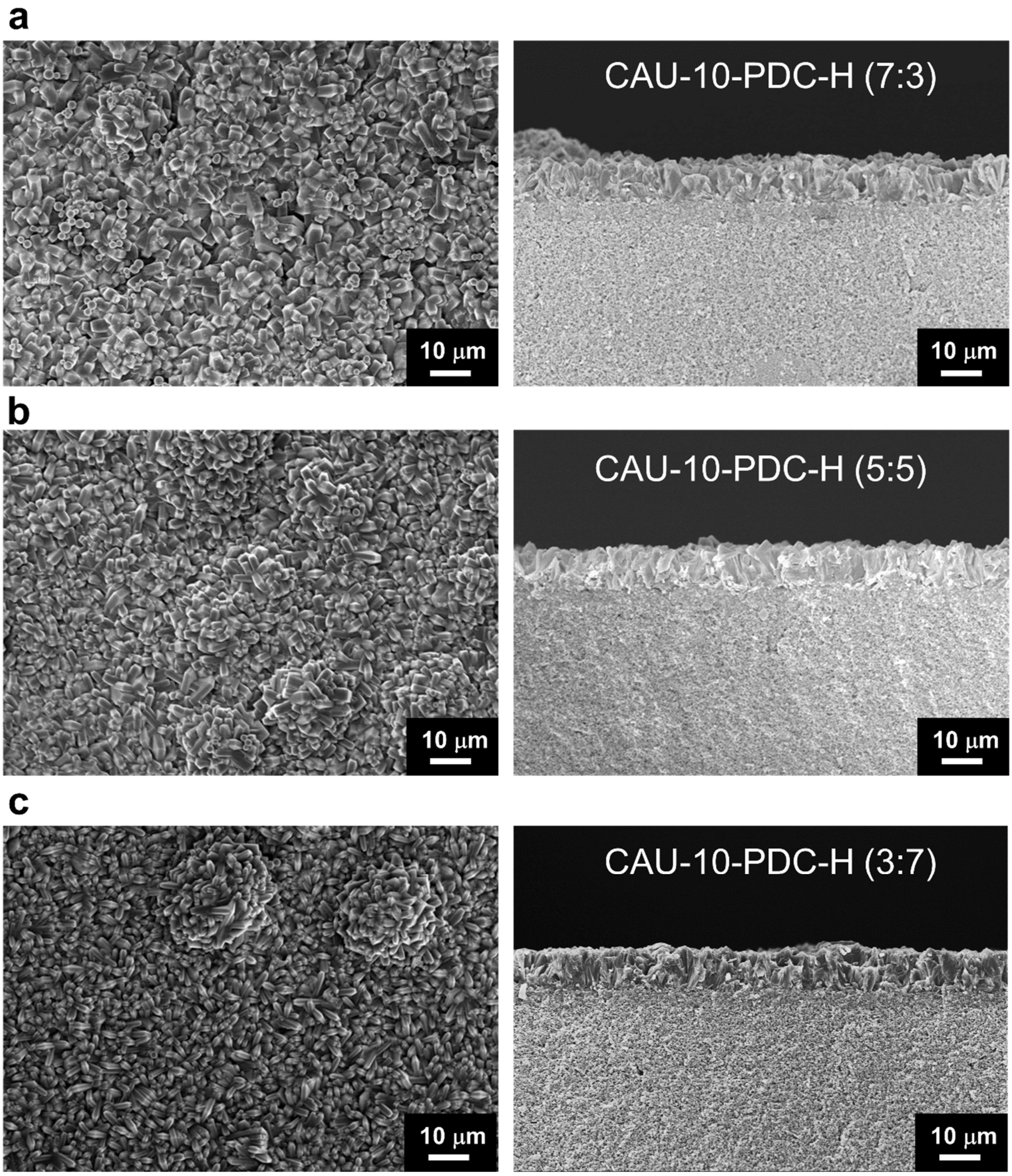

Supplementary Fig. 5. Top-view (left) and cross-sectional (right) SEM images of **a** CAU-10-PDC-H (7:3), **b** (5:5), and **c** (3:7) membranes following secondary growth.

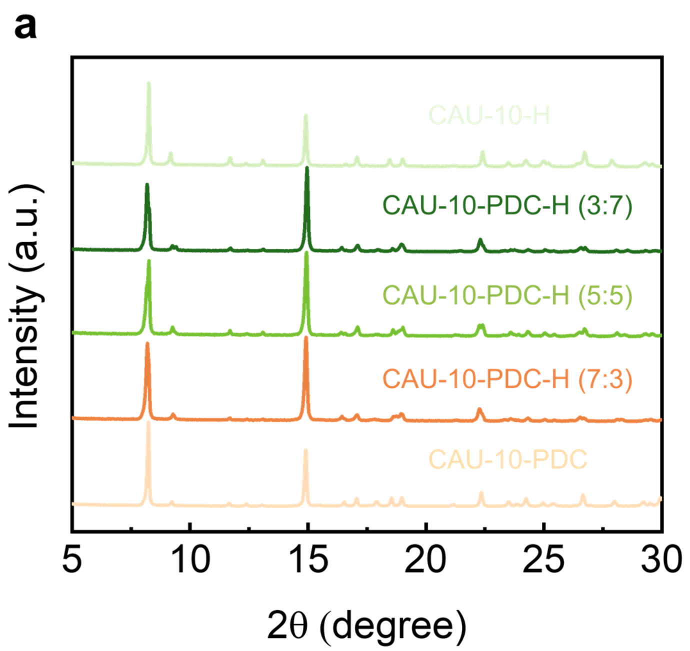

Supplementary Fig. 6. XRD patterns of CAU-10-PDC-H **a** powders and **b** membranes obtained using in-house X-ray source at 1.5418 Å.

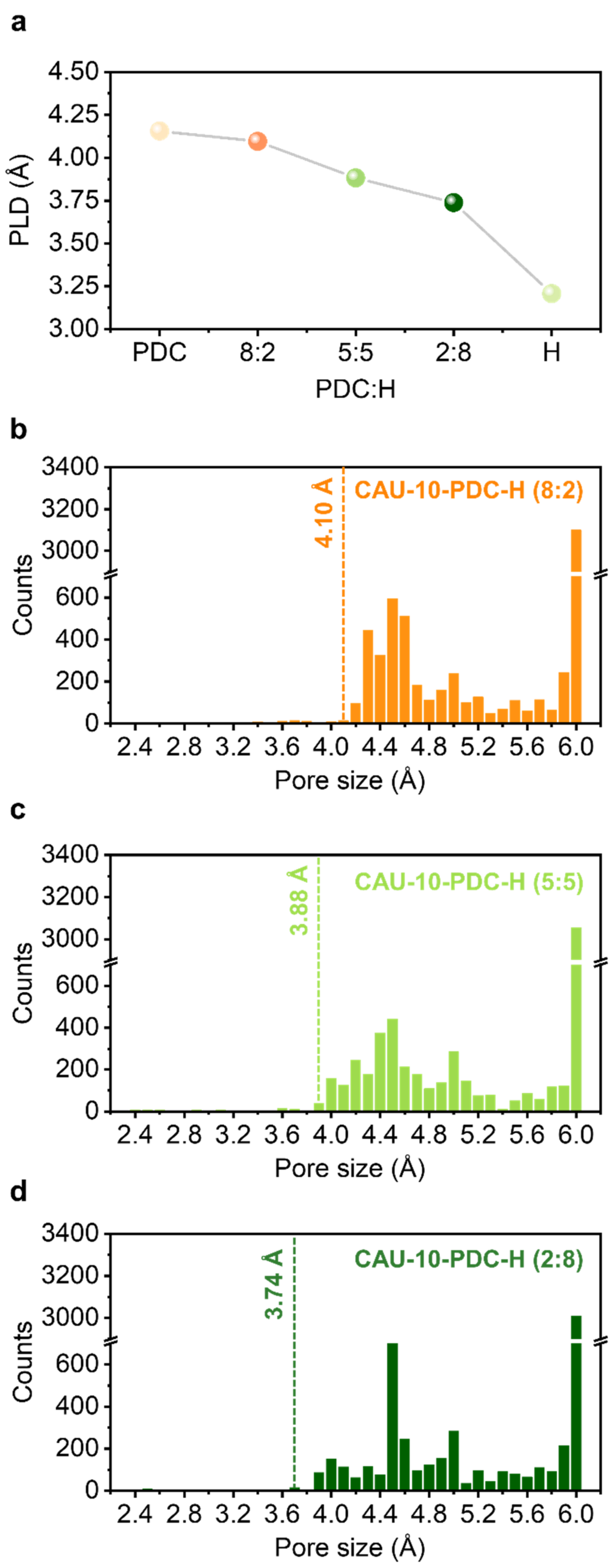

Supplementary Fig. 7 **a** Pore-limiting diameter and **b-d** pore size distribution of CAU-10-PDC-H structures obtained from Zeo++. Dash lines indicate the PLD for each structure. The PLDs of CAU-10-PDC and CAU-10-H were obtained from our previous reports[1,2].

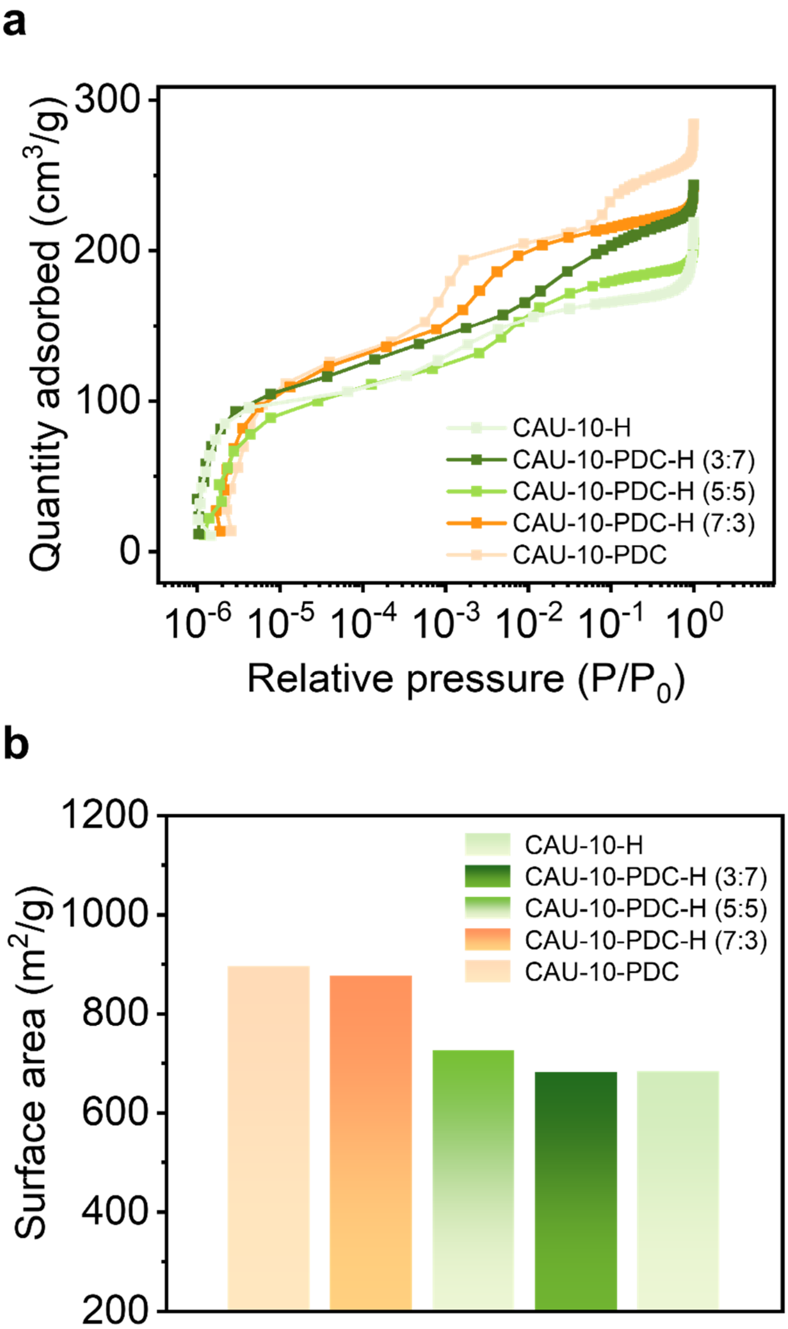

Supplementary Fig. 8 a Nitrogen adsorption isotherm measured at 77 K and **b** surface area obtained using BET+ESW method for pure CAU-10-PDC, CAU-10-H, and CAU-10-PDC-H powders.

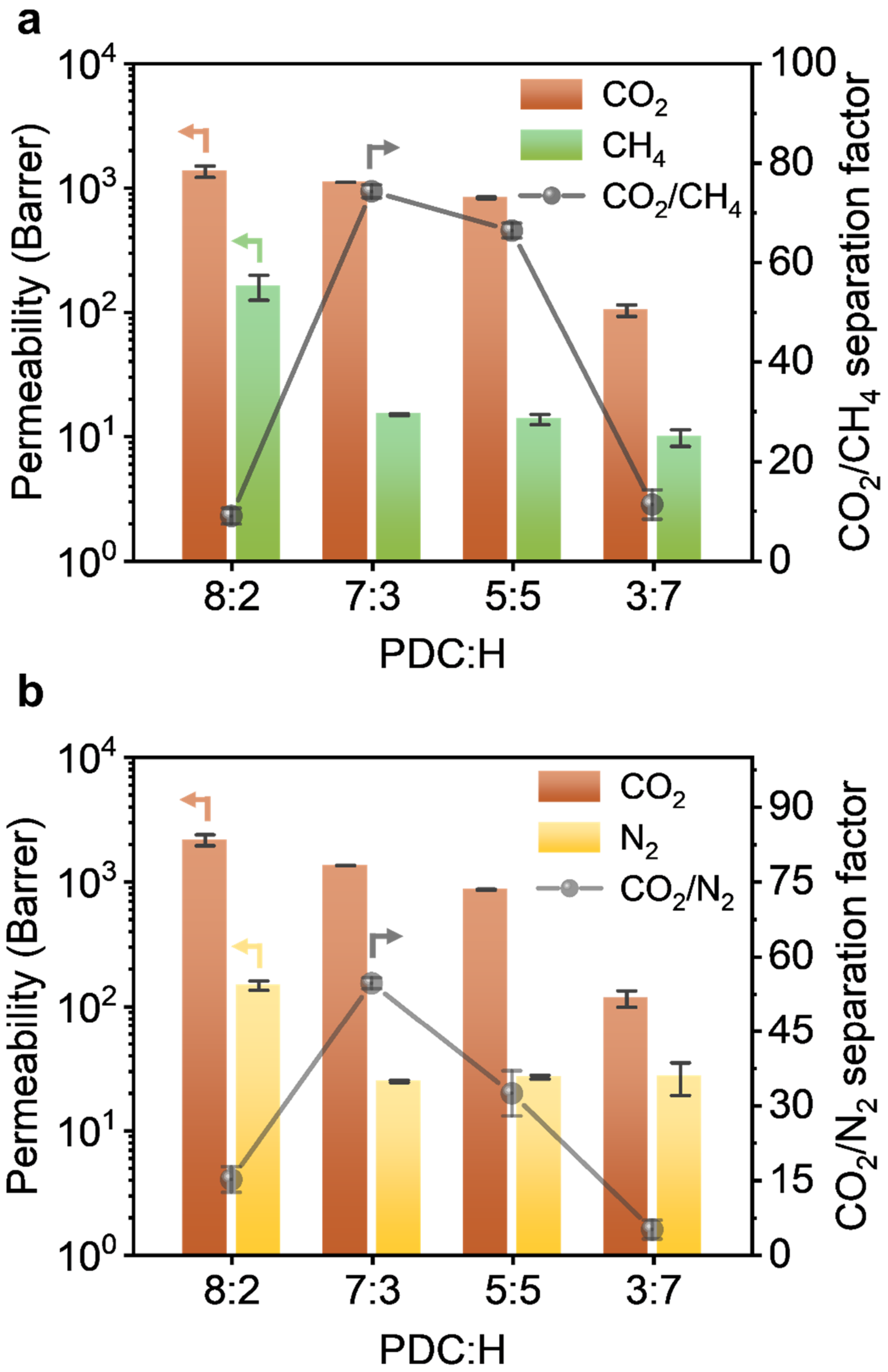

Supplementary Fig. 9 Mixed-gas permeability and separation factor of CAU-10-PDC-H membranes under feed pressure of 2 bar at 35°C for **a** CO_2/CH_4 and **b** CO_2/N_2, where the binary-gas feed is in a molar ratio of 50:50. The results presented in **a** and **b** represent the average performance of the three most effective membranes, based on a sample size of fewer than 10.

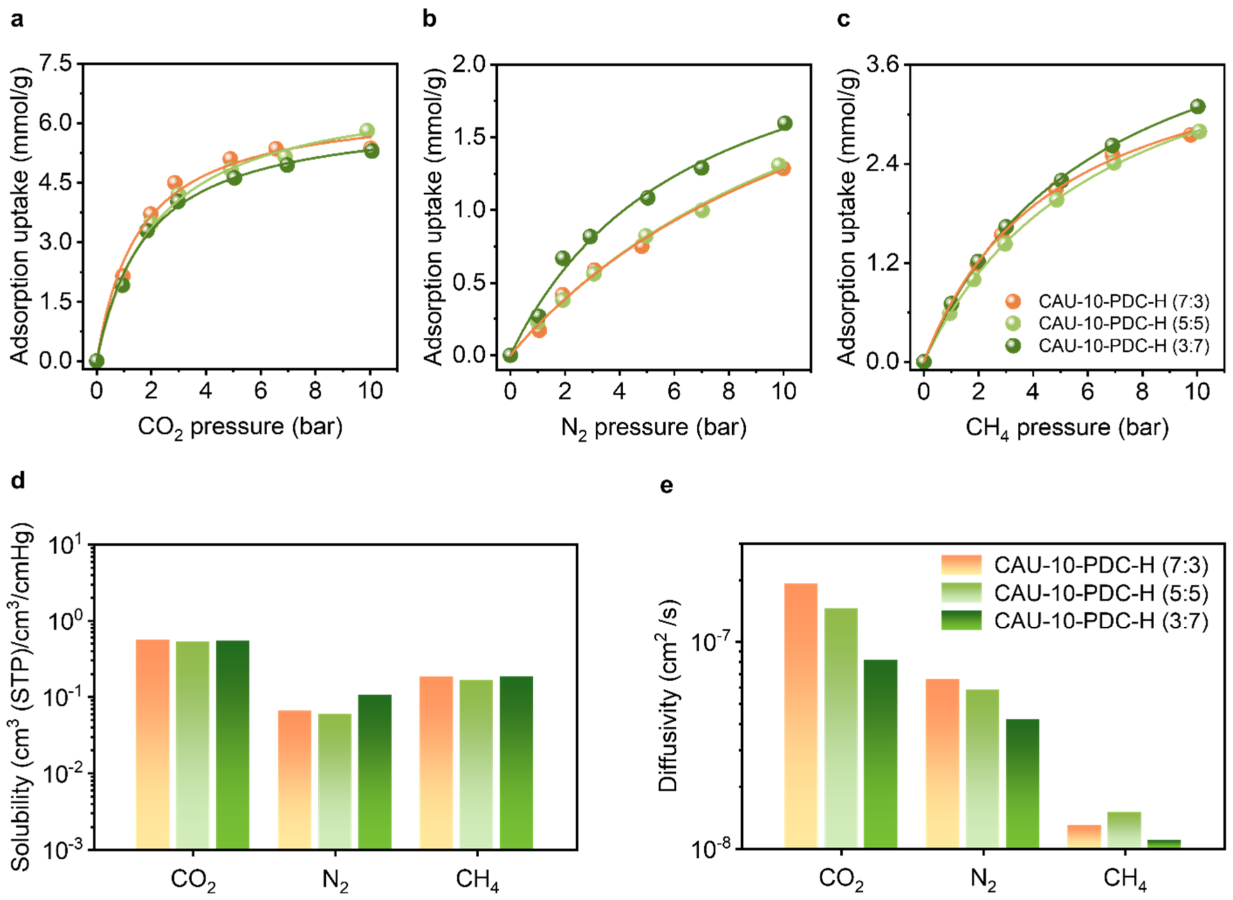

Supplementary Fig. 10. Compared single-component adsorption isotherms of **a** CO_2, **b** N_2, and **c** CH_4, and **d** solubility and **e** diffusivity of CO_2, N_2, and CH_4 for various CAU-10-PDC-H ratios under 308 K. Solid lines indicate the curve fitted using Langmuir model.

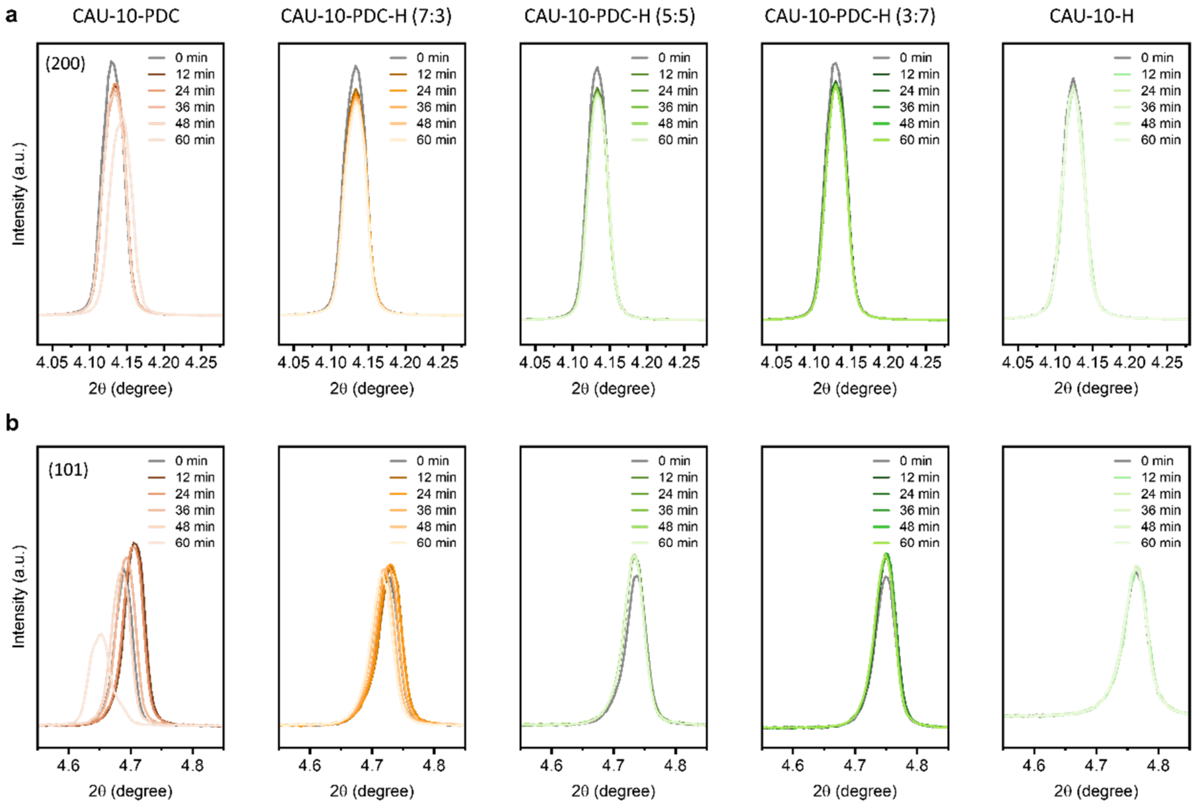

Supplementary Fig. 11. Time-resolved XRD patterns for **a** (200) and **b** (101) diffraction peaks of CAU-10-PDC, CAU-10-H, CAU-10-PDC-H (7:3), (5:5), and (3:7) exposed to CH_4 at 2 bar and 35°C obtained using synchrotron X-ray source at 0.77489 Å.

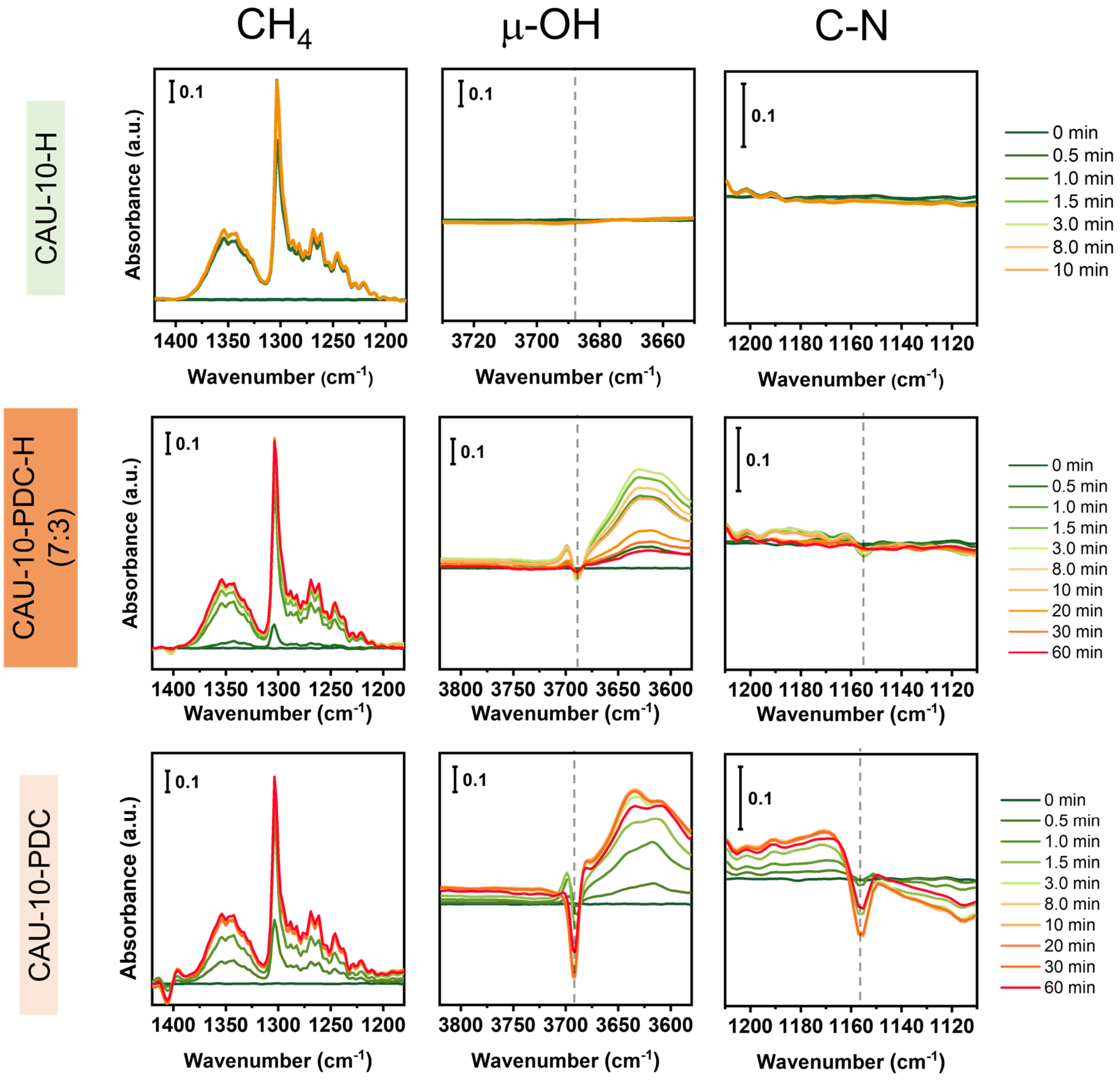

Supplementary Fig. 12. *In situ* DRIFT spectra of CAU-10-H, CAU-10-PDC-H (7:3) and CAU-10-PDC exposed to CH_4. The dashed lines indicate the absorption peak of corresponding bonds (μ-OH or C-N) before the gas exposure. A red and a blue shift are found in the absorption of μ-OH and C-N in CAU-10-PDC, respectively.

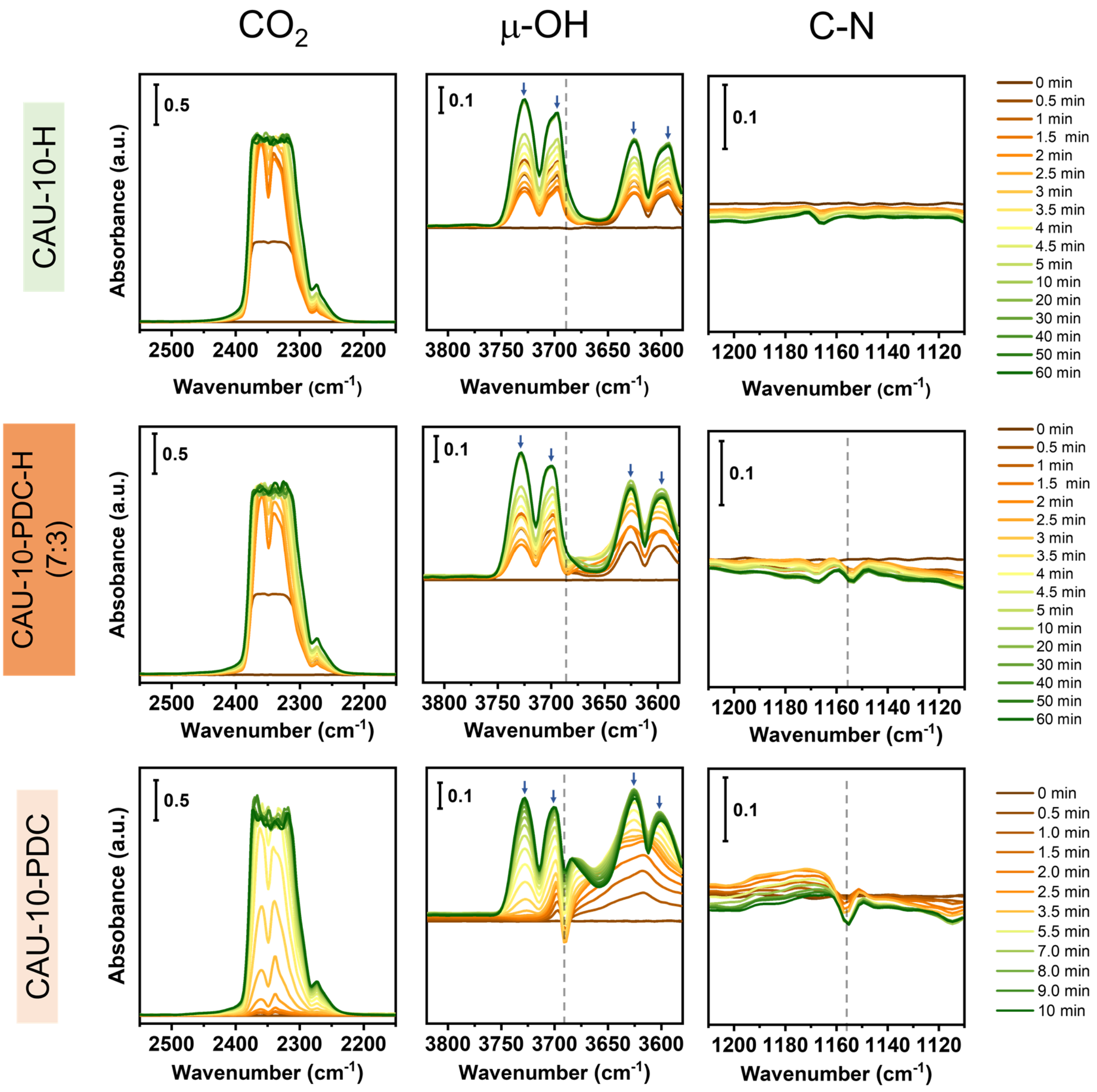

Supplementary Fig. 13. *In situ* DRIFT spectra of CAU-10-H, CAU-10-PDC-H (7:3) and CAU-10-PDC exposed to CO_2. Dashed lines indicate the absorption peak of μ-OH or C-N. In the middle column, the absorptions at 3727, 3698, 3625, and 3594 cm^{-1} are attributed to the adsorbed CO_2, as indicated by the arrows. Red and blue shifts are found in the absorptions of μ-OH and C-N in the CAU-10-PDC, respectively.

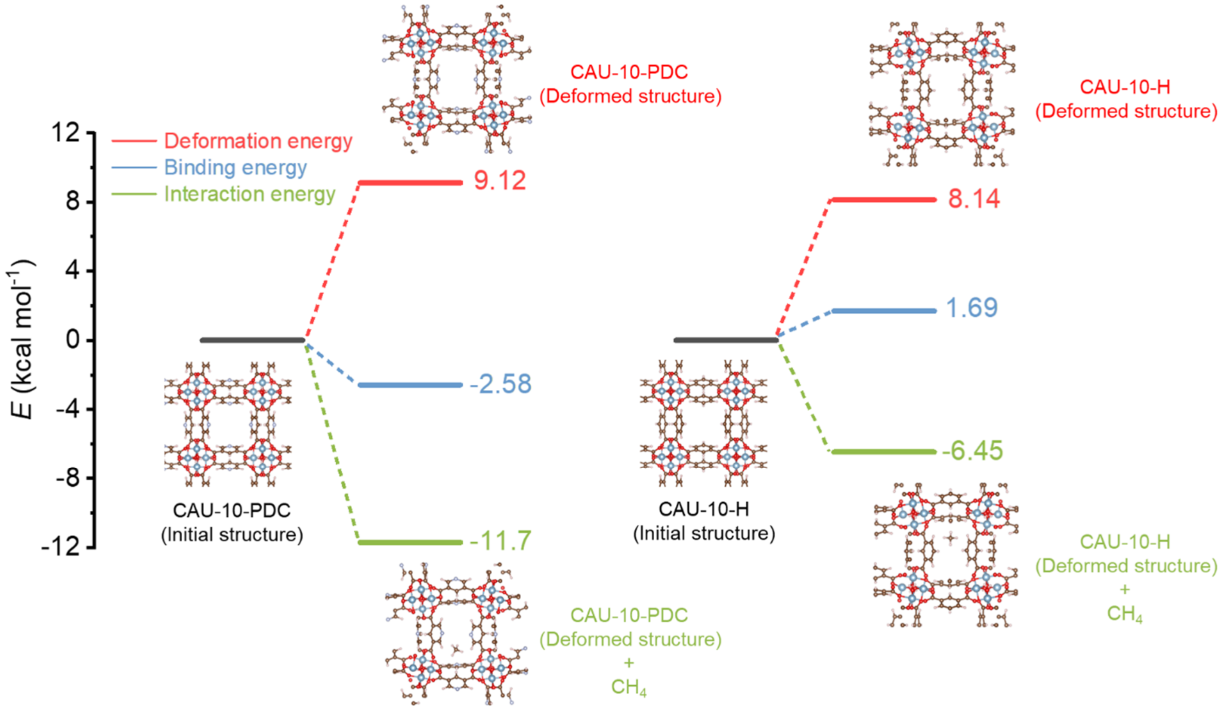

Supplementary Fig. 14 Energy decomposition analysis (EDA) and structural deformation of CAU-10-PDC and CAU-10-H upon adsorption of methane.

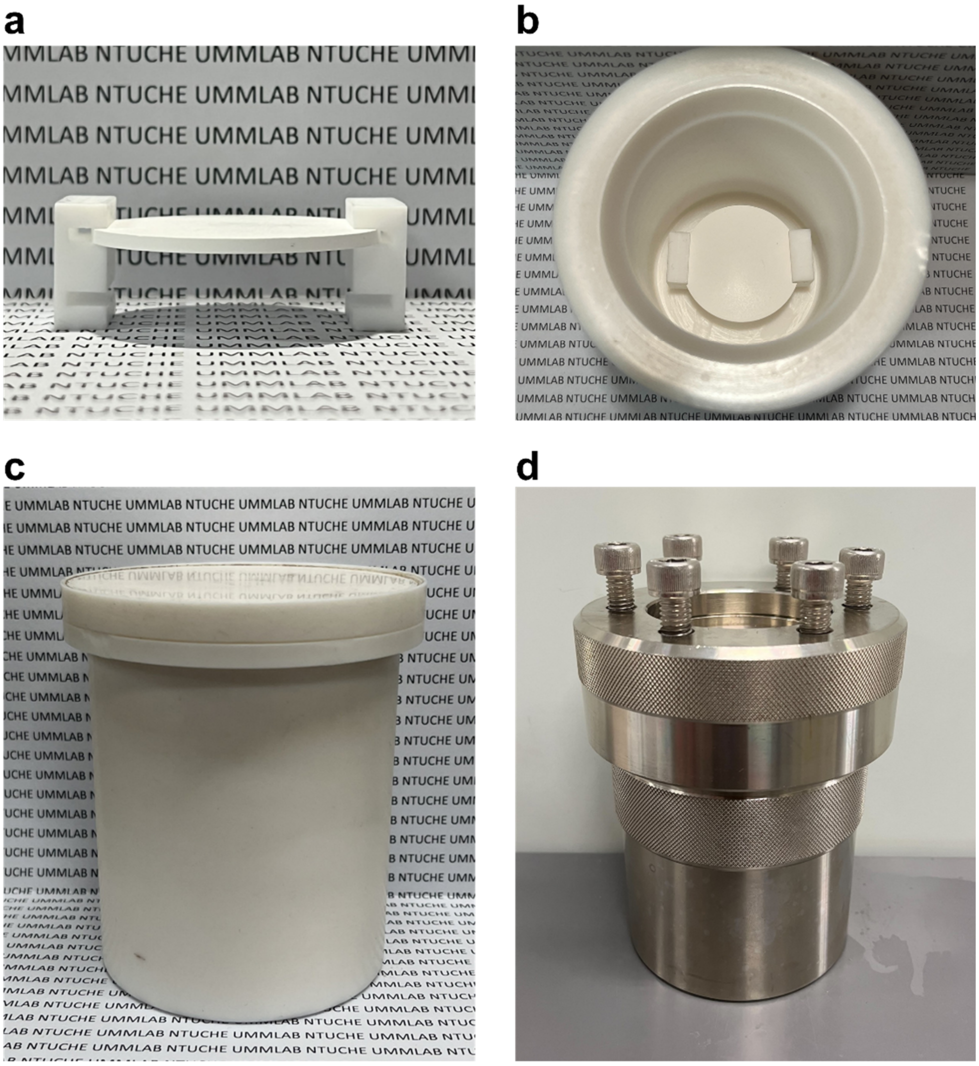

Supplementary Fig. 15. Photographic images showing the **a** substrate with Teflon holders and **b-d** the reaction setup used for secondary growth of a CAU-10-PDC-H membrane.

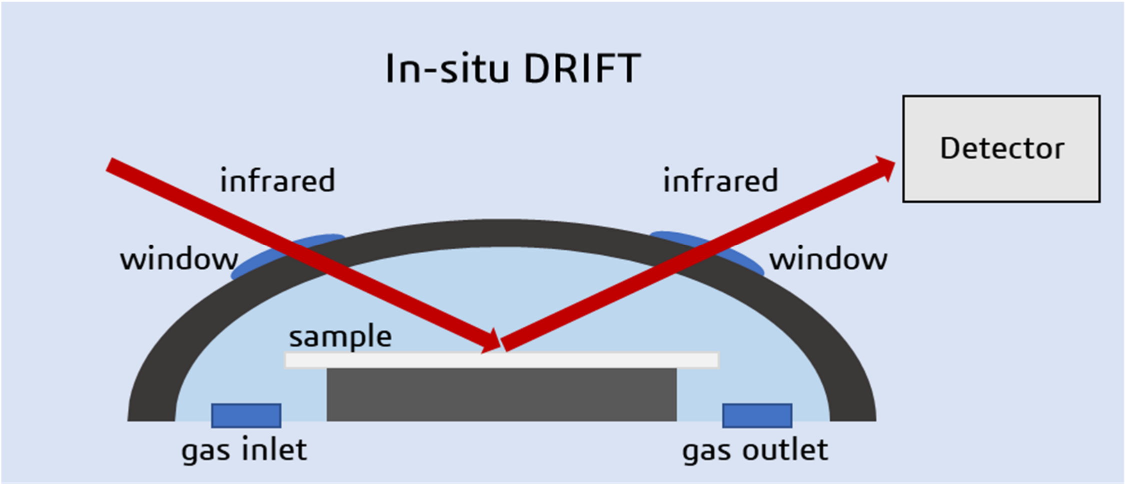

Supplementary Fig. 16. Schematic illustration of setup for *in situ* DRIFT studies on the MOF membrane samples.

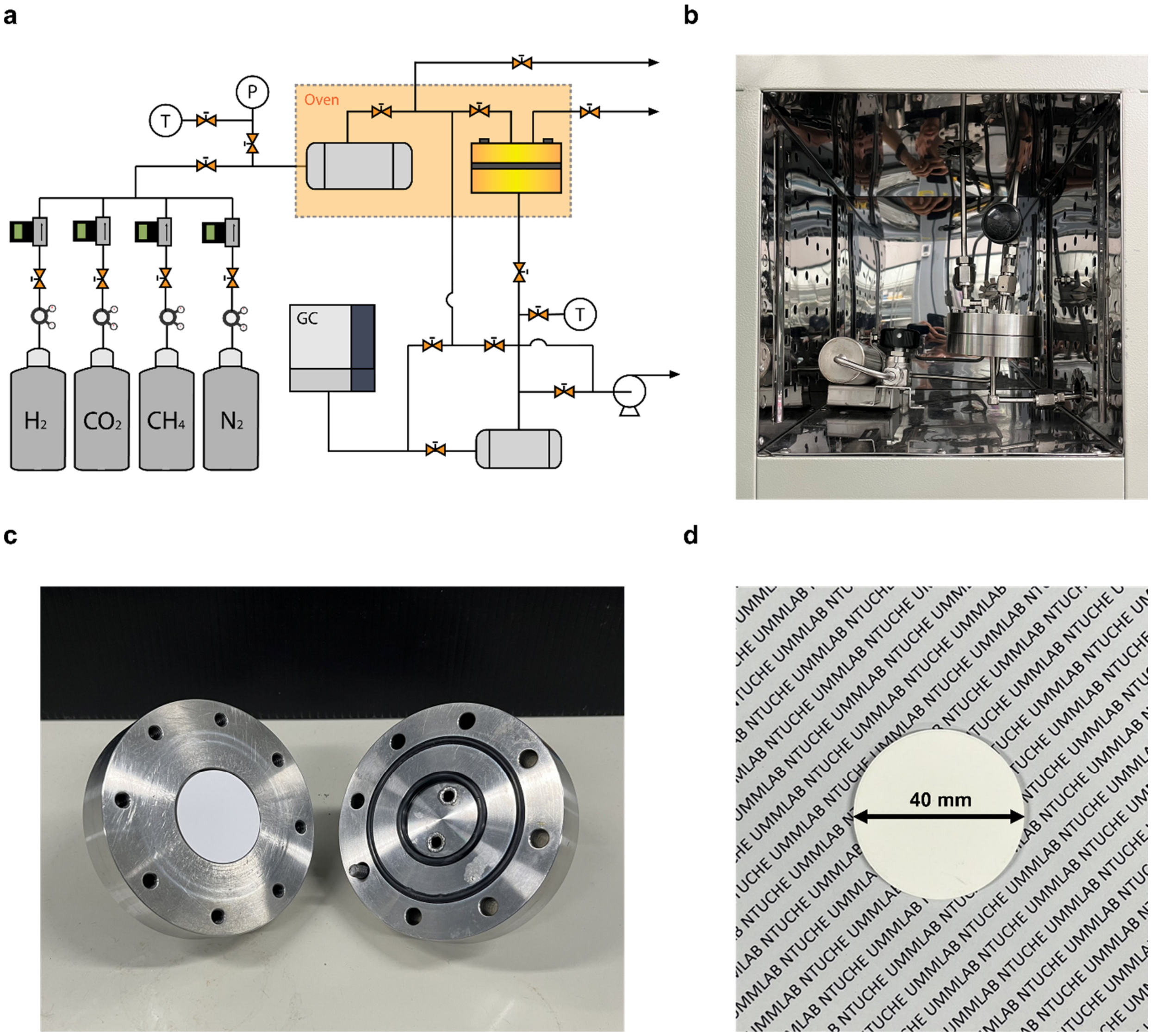

Supplementary Fig. 17. **a** Schematic illustration of setup for gas permeation system used in this study. The downstream mass flow rate was monitored using a pressure transducer to measure changes in pressure (the constant-volume method), and upstream or downstream composition was determined using GC. Photographic images showing the **b** membrane cell setup in gas permeation system, **c** inside of the membrane cell, and **d** α-alumina substrate.

Supplementary Tables

Supplementary Table 1 Molar percentage of the PDC linker in various CAU-10-PDC-H samples characterized by different methods.

	Powder Samples			Membrane Samples
	FTIR	NMR	EA	FTIR
CAU-10-PDC-H (7:3)	80.8	69.1	70.4	65.3
CAU-10-PDC-H (5:5)	54.9	48.5	52.1	43.6
CAU-10-PDC-H (3:7)	28.7	28.5	28.2	16.8

Supplementary Table 2 The permeability of various CAU-10-PDC-H membranes in single-gas measurement. All the membranes prepared in the work represented an average thickness of 10 μm.

Single gas						
Sample	P_{H2} (Barrer)	P_{CO2} (Barrer)	P_{N2} (Barrer)	P_{CH4} (Barrer)	CO_2/N_2 selectivity (-)	CO_2/CH_4 selectivity (-)
CAU-10-PDC-H (7:3)	1310.0 ± 4.1	1075.5 ± 8.8	42.9 ± 0.8	23.3 ± 0.2	25.1 ± 0.3	46.1 ± 0.8
CAU-10-PDC-H (5:5)	1168.5 ± 37.2	760.1 ± 11.4	34.9 ± 0.5	24.5 ± 0.1	21.8 ± 0.4	31.0 ± 0.4
CAU-10-PDC-H (3:7)	552.5 ± 80.3	436.2 ± 14.7	43.7 ± 0.6	20.0 ± 0.9	10.0 ± 0.5	21.8 ± 0.2

Note: The results presented in the above table represent the average performance of the three most effective membranes, based on a sample size of fewer than 10.

Supplementary Table 3 The permeance of various CAU-10-PDC-H membranes in single-gas measurement.

Single gas						
Sample	P_{H2} (10^{-8} mol m^{-2} s^{-1} Pa^{-1})	P_{CO2} (10^{-8} mol m^{-2} s^{-1} Pa^{-1})	P_{N2} (10^{-10} mol m^{-2} s^{-1} Pa^{-1})	P_{CH4} (10^{-10} mol m^{-2} s^{-1} Pa^{-1})	CO_2/N_2 selectivity (-)	CO_2/CH_4 selectivity (-)
CAU-10-PDC-H (7:3)	2.9 ± 0.1	2.4 ± 0.1	9.5 ± 0.2	5.2 ± 0.1	25.1 ± 0.3	46.1 ± 0.8
CAU-10-PDC-H (5:5)	3.0 ± 0.1	1.9 ± 0.1	8.9 ± 0.1	6.28 ± 0.1	21.8 ± 0.4	31.0 ± 0.4
CAU-10-PDC-H (3:7)	1.6 ± 0.4	1.20 ± 0.1	12.1 ± 1.8	5.49 ± 0.5	10.0 ± 0.5	21.8 ± 0.2

Note: The results presented in the above table represent the average performance of the three most effective membranes, based on a sample size of fewer than 10.

Supplementary Table 4 The permeability of various CAU-10-PDC-H membranes in mixed-gas measurement. All the membranes prepared in the work represented an average thickness of 10 μm.

Mixed gas						
Sample	P_{CO_2} (Barrer)	P_{N_2} (Barrer)	CO_2/N_2 separation factor (-)	P_{CO_2} (Barrer)	P_{CH_4} (Barrer)	CO_2/CH_4 separation factor (-)
CAU-10-PDC-H (8:2)	2176.2 ± 220.1	148.2 ± 12.7	14.9 ± 2.6	1359.6 ± 143.3	162.2 ± 36.9	8.7 ± 1.6
CAU-10-PDC-H (7:3)	1359.7 ± 6.1	25.0 ± 0.5	54.5 ± 1.1	1111.1 ± 0.3	15.0 ± 0.3	74.2 ± 1.3
CAU-10-PDC-H (5:5)	869.3 ± 12.8	27.0 ± 1.3	32.3 ± 1.5	834.3 ± 6.7	13.8 ± 0.9	60.9 ± 4.6
CAU-10-PDC-H (3:7)	116.8 ± 17.7	27.2 ± 8.0	4.8 ± 1.9	104.0 ± 11.1	9.9 ± 1.5	11.0 ± 2.9

Note: The results presented in the above table represent the average performance of the three most effective membranes, based on a sample size of fewer than 10.

Supplementary Table 5 The permeance of various CAU-10-PDC-H membranes in mixed-gas measurement.

Mixed gas						
Sample	P_{CO_2} (10^{-8} mol m^{-2} s^{-1} Pa^{-1})	P_{N_2} (10^{-10} mol m^{-2} s^{-1} Pa^{-1})	CO_2/N_2 separation factor (-)	P_{CO_2} (10^{-8} mol m^{-2} s^{-1} Pa^{-1})	P_{CH_4} (10^{-10} mol m^{-2} s^{-1} Pa^{-1})	CO_2/CH_4 separation factor (-)
CAU-10-PDC-H (8:2)	6.3± 1.1	43.0 ±6.5	14.9 ± 2.6	3.6 ± 0.4	43.1 ±11.2	8.7 ± 1.6
CAU-10-PDC-H (7:3)	3.5 ± 0.1	6.5 ± 0.1	54.5 ± 1.1	2.5 ± 0.1	3.3 ± 0.1	74.2 ± 1.3
CAU-10-PDC-H (5:5)	2.2 ± 0.1	6.9 ± 0.3	32.3 ± 1.5	2.1 ± 0.1	3.5 ± 0.2	60.9 ± 4.6
CAU-10-PDC-H (3:7)	0.4 ± 0.1	8.8 ±2.2	4.8 ± 1.9	0.4 ± 0.1	3.3 ± 0.5	11.0 ± 2.9

Note: The results presented in the above table represent the average performance of the three most effective membranes, based on a sample size of fewer than 10.

Supplementary Table 6 Reported membranes for gas separation in Figs. 3e and f.

Membrane material	CO_2 Permeability (Barrer)	CO_2/CH_4 Selectivity (-)	Ref.
CAU-10-PDC	[21]	[62]	1
CAU-10-H	508 [220]	95 [50]	2
IRMOF-1	6,269 [10,657]	1.09 [328]	3
ZIF-94	[28.4]	[38]	4
ZIF-8-RHT	77.2 [59.1]	28.8 [24.7]	5
ZIF-62-glass	724 [896]	26 [36]	6
CAU-1	9857	14.8	7
$Co_3(HCOO)_6$	73,925 [64,725]	5.4 [12.6]	8
sod-ZMOF	70.52 [54.51]	3.6 [4]	9
Bio-MOF-13	[120,370]	[3.8]	10
Bio-MOF-14	[161,529]	[3.5]	10
ZIF-69	2,817.91 [12,214.93]	2.7 [4.6]	11
UiO-66	208.96 [435.67]	12.5 [9.3]	12
CAU-10-PDC-H (7:3)	1,075.5 [1,111.1]	46.1 [74.2]	This work
CAU-10-PDC-H (5:5)	760.1 [834.3]	31.0 [60.9]	This work
CAU-10-PDC-H (3:7)	436.2 [104.0]	21.8 [11.0]	This work
**Membrane material**	**CO_2 Permeability (Barrer)**	**CO_2/N_2 Selectivity (-)**	**Ref.**
CAU-10-H	494	41	2
IRMOF-1	6,269 [8,609]	0.8 [410]	3
ZIF-8-RHT	66	37	5
ZIF-62-glass	2,047 [2,602]	23 [34]	6
CAU-1	11,642 [11,642]	26 [23]	7
sod-ZMOF	70	9	9
ZIF-69	2,818 [12,346]	2 [6]	11
UiO-66	209 [246]	31 [21]	12
MMOF	54	5	13
MOF-5	[18,806]	[70]	14
CAU-10-PDC-H (7:3)	1,075.5 [1359.7]	25.1 [54.5]	This work
CAU-10-PDC-H (5:5)	760.1 [869.3]	21.8 [32.3]	This work
CAU-10-PDC-H (3:7)	436.2 [116.8]	10.0 [4.8]	This work

[ ]: Mixed-gas separation

Supplementary Table 7 The FTIR peak positions of the functional groups of CAU-10-PDC computed by DFT.

Functional group	Peak position with original structure (cm^{-1})	Peak position with deformed structure (cm^{-1})	Peak shift between the two structures (cm^{-1})
μ-OH	3,766.1	3,599.2	-166.9
C-N	1,151.3	1,162.9	11.6

Supplementary Table 8 Peak assignments for DRIFT spectra shown in Fig. 6c and. Supplementary Figs. 12 and 13.

Functional group	Peak position before exposure to CH_4 or CO_2 (cm^{-1})	Peak position after exposure to CH_4 or CO_2 (cm^{-1})	Ref.
C-N	1,157	1,200-1,170	15
μ-OH	3,689	3,675-3,590	16

Supplementary References

1 Chang, C.-K. *et al.* Conformational-change-induced selectivity enhancement of CAU-10-PDC membrane for H_2/CH_4 and CO_2/CH_4 separation. *J. Membr. Sci. Lett.* **1**, 100005, (2021).

2 Chiou, D. S. *et al.* Highly CO_2 Selective Metal–Organic Framework Membranes with Favorable Coulombic Effect. *Adv. Funct. Mater.*, 2006924, (2020).

3 Rui, Z., James, J. B., Kasik, A. & Lin, Y. S. Metal-organic framework membrane process for high purity CO_2 production. *AIChE J.* **62**, 3836-3841, (2016).

4 Cacho-Bailo, F., Etxeberría-Benavides, M., Karvan, O., Téllez, C. & Coronas, J. Sequential amine functionalization inducing structural transition in an aldehyde-containing zeolitic imidazolate framework: application to gas separation membranes. *CrystEngComm* **19**, 1545-1554, (2017).

5 Babu, D. J. *et al.* Restricting Lattice Flexibility in Polycrystalline Metal-Organic Framework Membranes for Carbon Capture. *Adv. Mater.* **31**, 1900855, (2019).

6 Wang, Y. *et al.* A MOF Glass Membrane for Gas Separation. *Angew. Chem. Int. Ed. Engl.* **59**, 4365-4369, (2020).

7 Yin, H. *et al.* A highly permeable and selective amino-functionalized MOF CAU-1 membrane for CO_2-N_2 separation. *Chem. Commun.* **50**, 3699-3701, (2014).

8 Zou, X. *et al.* $Co_3(HCOO)_6$ microporous metal-organic framework membrane for separation of CO_2/CH_4 mixtures. *Chem. Eur. J.* **17**, 12076-12083, (2011).

9 Al-Maythalony, B. A. *et al.* Quest for anionic MOF membranes: continuous sod-ZMOF membrane with CO_2 adsorption-driven selectivity. *J. Am. Chem. Soc.* **137**, 1754-1757, (2015).

10 Xie, Z., Li, T., Rosi, N. L. & Carreon, M. A. Alumina-supported cobalt–adeninate MOF membranes for CO_2/CH_4 separation. *J. Mater. Chem. A* **2**, 1239-1241, (2014).

11 Liu, Y., Zeng, G., Pan, Y. & Lai, Z. Synthesis of highly *c*-oriented ZIF-69 membranes by secondary growth and their gas permeation properties. *J. Membr. Sci.* **379**, 46-51, (2011).

12 Rong, R., Sun, Y., Ji, T. & Liu, Y. Fabrication of highly CO_2/N_2 selective polycrystalline UiO-66 membrane with two-dimensional transition metal dichalcogenides as zirconium source via tertiary solvothermal growth. *J. Membr. Sci.* **610**, 118275, (2020).

13 Ranjan, R. & Tsapatsis, M. Microporous Metal Organic Framework Membrane on Porous Support Using the Seeded Growth Method. *Chem. Mater.* **21**, 4920-4924, (2009).

14 Zhao, Z., Ma, X., Kasik, A., Li, Z. & Lin, Y. S. Gas Separation Properties of Metal Organic Framework (MOF-5) Membranes. *Ind. Eng. Chem. Res.* **52**, 1102-1108, (2012).

15 Ibrahim, I., Yunus, S. & Hashim, A. Relative Performance of Isopropylamine, Pyrrole and Pyridine as Corrosion Inhibitors for Carbon Steels in Saline Water at Mildly Elevated Temperatures. *Int. J. Sci. Eng. Res.* **4**, (2013).

16 Kim, H. *et al.* Water harvesting from air with metal-organic frameworks powered by natural sunlight. *Science* **356**, 430-434, (2017).
